# ATP7A-mediated copper ion efflux reprogrammes tumour immunity and promotes cisplatin resistance in cervical cancer

**DOI:** 10.1038/s41419-026-09082-5

**Published:** 2026-07-10

**Authors:** Shuangjia Pan, Qianqian Wu, Hejing Liu, Yujia Zhou, Jiayi Ruan, Yuqing Jin, Lingxiao Huang, Jian’an Zhang, Huihui Ji, Xueqiong Zhu

**Affiliations:** 1 Zhejiang Provincial Clinical Research Center for Gynecological Diseases, Department of Obstetrics and Gynecology, The Second Affiliated Hospital of Wenzhou Medical University, Wenzhou, Zhejiang China; 2 Department of Obstetrics and Gynecology, Wenzhou People’s Hospital, Wenzhou, Zhejiang China

**Keywords:** Cancer, Cervical cancer

## Abstract

Cisplatin resistance is a major obstacle to effective chemotherapy in patients with cervical cancer and is associated with an immunosuppressive tumour microenvironment. Copper ions are essential trace elements that regulate redox and immune balance. However, the connections among copper ions, cisplatin resistance and immune regulation remain unclear. Here, higher serum copper levels correlated with increased pan-cancer mortality. Integrative transcriptomic and single-cell RNA sequencing analyses revealed lower copper-related transcriptional activity in CD8⁺ T cells from cervical cancer tissues. Multiomics analysis revealed that ATPase copper transporting alpha (ATP7A) expression was upregulated in cisplatin-resistant patients compared with sensitive patients and correlated with poor survival. ATP7A knockdown increased sensitivity to cisplatin and promoted cuproptosis, whereas ATP7A overexpression increased cisplatin resistance and impaired CD8⁺ T cells through copper ion efflux. Furthermore, excess copper ions directly bind to the lymphocyte-specific protein tyrosine kinase (LCK) activation loop (Tyr394), impairing T-cell receptor (TCR) signalling in CD8⁺ T cells. Sex-determining region Y-box 21 (SOX21) directly binds two conserved motifs within the ATP7A promoter (-372 bp and -245 bp) and enhances its transcriptional activity. These results revealed a SOX21/ATP7A/copper ion axis connecting copper ion efflux with immune suppression and cisplatin resistance. Targeting ATP7A or copper ion efflux may restore CD8⁺T-cell activity and enhance the efficacy of cisplatin for the treatment of cervical cancer.

## Introduction

Cisplatin resistance in cervical cancer cells acts as a key driver of treatment failure and poor prognosis, and this phenomenon represents a major clinical challenge across multiple malignancies, including lung, ovarian, head and neck, and breast cancers [1]. Beyond intrinsic mechanisms such as enhanced DNA repair, elevated drug efflux and redox adaptation, mounting evidence suggests that the tumour microenvironment (TME) plays a pivotal role in driving cisplatin resistance [2]. In particular, an immunosuppressive TME, in which CD8⁺ T cells lose their cytotoxic function, accelerates the establishment of cisplatin resistance [2, 3]. Conversely, cells resistant to cisplatin reprogram immune cells, impairing antigen presentation and weakening cytotoxic T-cell activation [4, 5]. This feedback loop links immune suppression with cisplatin resistance, promoting immune escape and persistent chemoresistance.

Copper (Cu), an essential trace element, plays critical roles in cellular metabolism, mitochondrial respiration and redox regulation [6]. Dysregulation of copper ion homeostasis is associated with various pathophysiological processes, including tumour progression, therapeutic resistance, and immune modulation [7, 8]. In addition, copper transporters play a critical role in preserving intracellular metal homeostasis and are closely linked to cisplatin resistance [9]. Reduced expression of the copper transporter 1/solute carrier family 31 member 1 (CTR1/SLC31A1) limits cisplatin uptake, whereas increased expression of the efflux transporters ATPase copper transporting alpha (ATP7A) and ATPase copper transporting beta (ATP7B) enhances drug sequestration and export [9, 10]. This phenomenon reduces the intracellular platinum concentration near nuclear DNA and drives transporter-mediated chemoresistance [9–11]. Cuproptosis refers to a type of programmed cell death triggered by excessive intracellular copper ion accumulation, characterized by mitochondrial stress and the aggregation of lipoylated proteins [12, 13]. Our previous study demonstrated that inducing cuproptosis in cervical cancer cells markedly increased their sensitivity to cisplatin [14]. Intracellular copper levels are tightly regulated by a broader copper transport network encompassing influx transporters (CTR1, copper transporter 2 (CTR2)), efflux transporters (ATP7A and ATP7B), and potentially leucine rich repeat containing 8 family member A (LRRC8A)-associated mechanisms, with ATP7A acting as a key efflux pump to maintain copper balance [15, 16]. Aberrant expression of the ATP7A protein is associated with various malignancies, such as lung and liver cancer, and is associated with cisplatin resistance and poor prognosis [17, 18]. However, the function of ATP7A in cisplatin resistance and immune suppression in cervical cancer remains unexplored, and the upstream mechanisms regulating the expression of ATP7A are still unclear.

Therefore, in this study, we hypothesized that ATP7A-mediated copper efflux promoted cisplatin resistance and immune suppression in cervical cancer by disrupting the copper balance and impairing CD8⁺ T-cell function. Using an integrated approach combining multiomics analysis and cellular and animal experiments, the role of ATP7A and copper in cisplatin-resistant cervical cancer was comprehensively explored. This work provides new biological insight into the relationship between cisplatin resistance and immune suppression.

## Results

### Serum copper levels and expression of the copper transporter ATP7A are associated with cervical cancer progression and cisplatin resistance

**Fig. 1:**
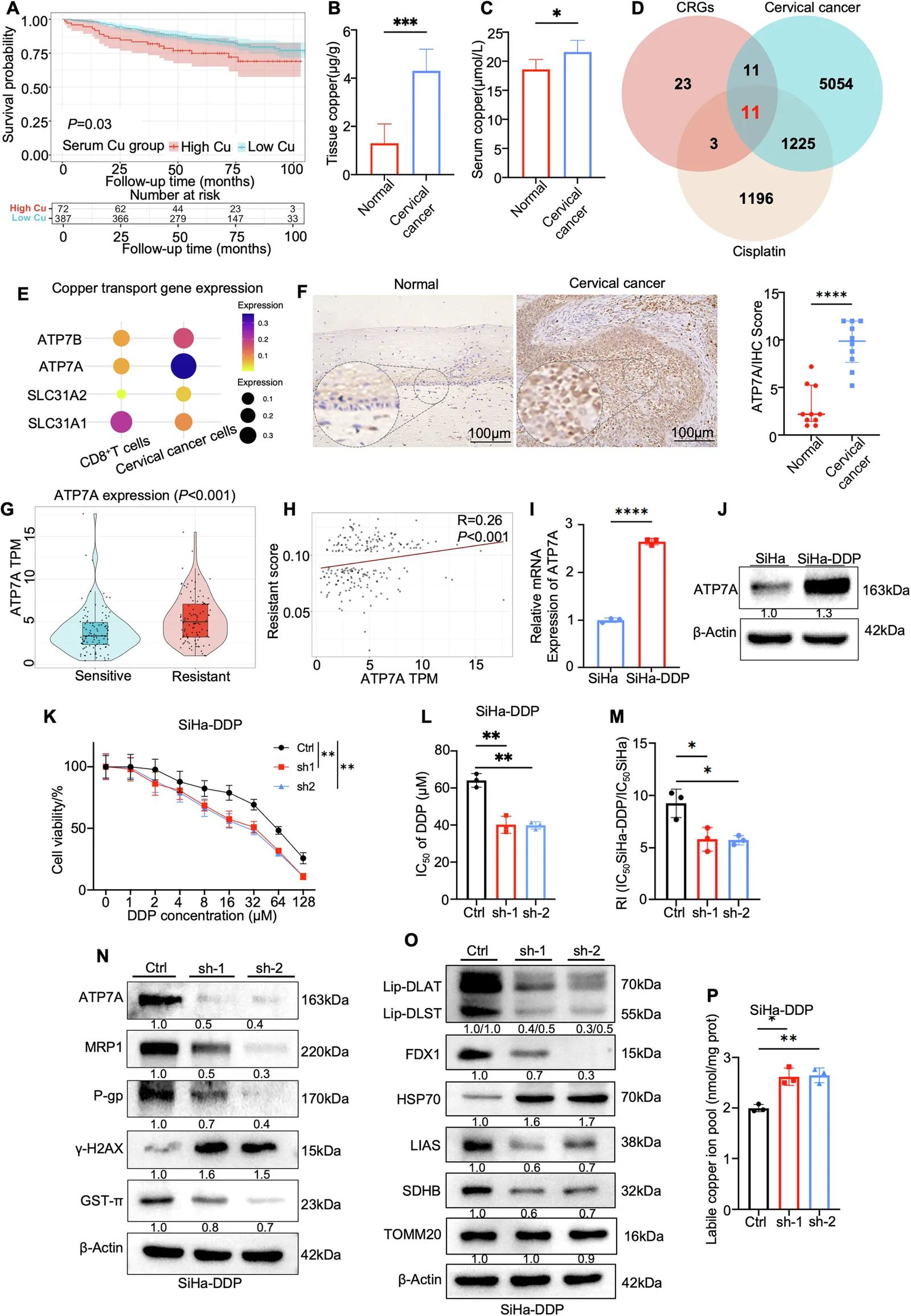
Analysis of copper levels and ATP7A expression in cervical cancer and their association with cisplatin resistance. **A** Weighted Kaplan-Meier survival analysis of the NHANES 2011-2016 cancer cohort (*n* = 459) stratified by the serum copper concentration. **B** Comparison of copper ion levels in normal cervical tissues/cervical cancer tissues between normal individuals and cervical cancer patients. **C** Comparison of serum copper ion levels between normal individuals and patients with cervical cancer. **D** Intersection analysis of cervical cancer-related genes, cuproptosis-related genes and cisplatin resistance genes. CGRs: cuproptosis-related genes. **E** Expression profiling of copper transporter genes (ATP7A, ATP7B, SLC31A1, and SLC31A2) across annotated cell types. **F** Immunohistochemical (IHC) staining of ATP7A in normal cervical epithelium and cervical cancer tissues and quantification of IHC scores. **G** Comparison of ATP7A expression between cisplatin-sensitive and -resistant tumours in the cervical cancer cohort. **H** Analysis of the correlation between ATP7A transcript levels and chemoresistance scores. **I** qRT-PCR analysis of relative ATP7A mRNA expression in SiHa and SiHa-DDP cells. **J** Western blot analysis of ATP7A protein expression in parental SiHa and SiHa-DDP cells. **K**-**M** Cell viability and drug sensitivity of SiHa-DDP cells treated with cisplatin (0, 1, 2, 4, 8, 16, 32, 64, or 128 µM) for 48 h determined by a CCK-8 assay. **N** Western blot analysis of ATP7A, MRP1, P-gp, γ-H2AX, and GST-π expression in SiHa-DDP cells after ATP7A knockdown. **O** Western blot analysis of the expression of cuproptosis-related proteins (Lip-DLAT, Lip-DLST, FDX1, LIAS, SDHB, TOMM20, and HSP70) in SiHa-DDP cells with ATP7A knockdown. **P** Measurement of the intracellular Cu²⁺ concentration in SiHa-DDP cells.

On the basis of data from the NHANES 2011-2016 pan-cancer cohort (*n* = 459), serum copper (LBDSCUSI) levels were dichotomized into high vs. low groups by using the maximally selected rank statistic (cutpoint = 22.14 µmol/L) (Table S2, Fig. S1A). Compared with the high-Cu group, the low-Cu group exhibited significantly superior overall survival, as shown by Kaplan-Meier curves, indicating that higher circulating copper levels were associated with worse survival (Fig. 1A). Furthermore, copper levels were notably higher in both the serum and tumour tissues of patients with cervical cancer than in those of normal controls (Fig. 1B, C). Intersection analysis of cervical cancer-related genes, cuproptosis-related genes and cisplatin resistance genes revealed 11 key genes associated with cervical cancer progression and cisplatin resistance (including ATP7A, ATP7B, SLC31A1, SLC31A2, GLS, CDKN2A, MAP2K1, SOD1, VEGFA, MAP2K2, and NFE2L2) (Fig. 1D). Moreover, analysis of the expression of copper transporter genes further revealed that ATP7A was the most highly expressed copper transporter in cervical cancer cells (Fig. 1E).

Further investigations were conducted to examine the function of ATP7A. Patients with high ATP7A expression had shorter overall survival, as demonstrated by Kaplan-Meier analysis (Fig. S1B). To explore the correlation between cervical cancer and ATP7A protein expression, the expression of ATP7A was analysed in ten cervical cancer tissues and ten normal cervical tissues by immunohistochemistry. The results revealed that ATP7A protein expression was notably higher in cervical cancer tissues than in normal cervical epithelium (Fig. 1F). Similarly, compared with normal cervical epithelial cells, cervical cancer cell lines presented significantly increased ATP7A protein expression (Fig. S1C). Collectively, these results indicated that ATP7A might be a key copper transporter associated with altered copper homeostasis, immunosuppressive remodelling of the tumour microenvironment, and potential cisplatin resistance in cervical cancer.

### ATP7A expression modulation affects cisplatin sensitivity in cervical cancer

To investigate the association between ATP7A expression and cisplatin resistance, patients in the CESC dataset were grouped as cisplatin-sensitive or cisplatin-resistant on the basis of model-predicted resistance scores (Fig. S1D). ROC analysis confirmed the strong predictive performance of both models, with AUCs of 0.938 (Elastic Net) and 0.925 (XGBoost) (Fig. S1E). ATP7A mRNA expression was elevated in the cisplatin-resistant cases (Fig. 1G) and positively correlated with the resistance score (Fig. 1H). A cisplatin-resistant cervical cancer cell line (SiHa-DDP) was successfully established, with a calculated resistance index (RI) of 6.64 (Fig. S1F, G). The western blot and qPCR results suggested that ATP7A expression was substantially increased in SiHa-DDP cells compared with parental SiHa cells (Fig. 1I, J). CCK-8 assays demonstrated that ATP7A knockdown markedly reduced the viability of both parental SiHa and SiHa-DDP cells (Fig. S2A–D). Consistent with these findings, the results of the colony formation assays revealed that silencing ATP7A expression significantly impaired cell proliferation in both cell lines compared with the control groups, while the degree of apoptosis notably increased (Fig. S2E–L).

Afterwards, SiHa and SiHa-DDP cells were exposed to increasing concentrations of cisplatin. ATP7A knockdown enhanced cisplatin sensitivity in both SiHa and SiHa-DDP cells (Fig. 1K, L, Fig. S3A, B), accompanied by a marked reduction in the drug RI (Fig. 1M). Silencing of ATP7A led to decreased expression of proteins linked to drug resistance (P-gp, GST-π, and MRP1) and increased γ-H2AX accumulation, indicating enhanced DNA damage (Fig. 1N). In contrast, ATP7A overexpression markedly promoted cisplatin resistance in SiHa and SiHa-DDP cells (Fig. S3C–F).

### Modulation of ATP7A expression affects cisplatin sensitivity via cuproptosis

Given that excessive intracellular copper accumulation triggers cuproptosis and that ATP7A functions as a major copper exporter, the potential involvement of ATP7A in cisplatin resistance via cuproptosis was further examined. ATP7A knockdown resulted in reduced expression of cuproptosis-related proteins (Lip-DLST, Lip-DLAT, FDX1, LIAS, and SDHB), as well as downregulated HSP70 protein expression, while TOMM20 protein expression showed no obvious change. Meanwhile, intracellular copper ion levels were significantly increased (Fig. 1O, P, Fig. S3G, H). These findings indicated that ATP7A silencing promoted intracellular copper accumulation and triggered cuproptosis. Importantly, treatment with the cuproptosis inhibitor TTM reversed the sensitization of ATP7A-silenced SiHa-DDP cells to cisplatin (Fig. S3I, J). Collectively, these results demonstrated that ATP7A knockdown reversed cisplatin resistance in cervical cancer cells by promoting intracellular copper accumulation and inducing cuproptosis.

### Cisplatin-resistant cervical cancer cells suppresses CD8^+^ T cells

**Fig. 2:**
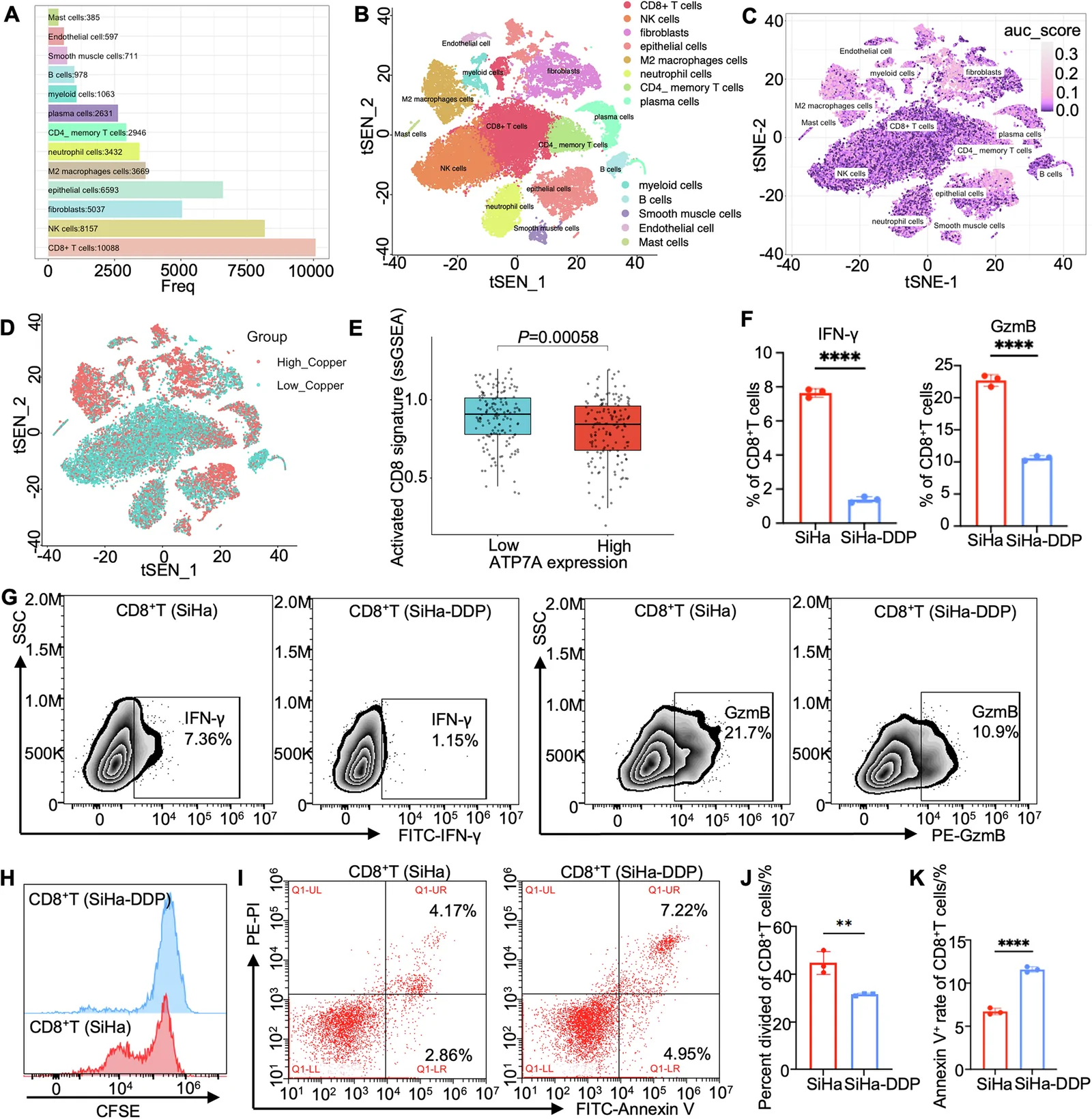
Cisplatin-resistant cervical cancer cells and their interaction with CD8^+^ T cells. **A** Cell-type distribution identified from integrated single-cell RNA-seq datasets of cervical cancer. **B** Integrated scRNA-seq analysis of 20,615 cells from 11 cervical cancer patients. **C** t-SNE visualization of AUCell-derived copper-related transcriptional activity across the tumour microenvironment. **D** AUCell-based scoring and visualization of high-copper and low-copper-activity groups across the cervical cancer microenvironment. **E** Analysis of the correlation between ATP7A expression in cervical cancer and the activated CD8⁺ T-cell gene signature. **F** Quantification of IFN-γ⁺ and GzmB⁺ CD8⁺ T-cell populations. **G** Flow cytometry analysis of intracellular IFN-γ and GzmB expression in CD8⁺ T cells cocultured with SiHa or SiHa-DDP cells. **H**, **I** CFSE-based proliferation and apoptosis assays of CD8⁺ T cells cocultured with SiHa or SiHa-DDP cells. **J**-**K** Quantification of CFSE-based cell proliferation and apoptosis.

To investigate the function of ATP7A and copper homeostasis in cervical cancer, multiple single-cell RNA sequencing datasets were integrated. After stringent quality control and normalization, a total of 20,671 cells from 11 cervical cancer patients and 29,630 highly variable genes were retained for analysis. Twenty discrete clusters, including CD8⁺ T cells, fibroblasts, epithelial cells, M2 macrophages, neutrophils, NK cells, CD4⁺ memory T cells, plasma cells, myeloid cells, B cells, smooth muscle cells, endothelial cells and mast cells, were identified and classified into 13 major cell types (Fig. 2A, B). AUCell-based scoring of copper-related gene sets revealed strong heterogeneity among different cell populations. Fibroblasts, M2 macrophages and myeloid cells displayed elevated copper-related activity, whereas CD8⁺ T cells and NK cells exhibited markedly lower scores (Fig. 2C). When the data were stratified by copper homeostasis-associated gene signature, effector T cells, such as CD8⁺ T cells, were predominantly distributed in the group with suppressed copper regulatory transcript levels (Fig. 2D). Immune infiltration analysis revealed that tumours with high ATP7A mRNA expression exhibited a lower percentage of CD8⁺ T cells (Fig. 2E). Moreover, ATP7A expression was inversely correlated with IFN-γ and granzyme B (GzmB) levels produced by CD8⁺ T cells (Fig. S4A, B), suggesting that high ATP7A expression had a suppressive effect on CD8⁺ T-cell cytotoxicity. These findings suggested that elevated ATP7A expression in cisplatin-resistant tumour cells might modulate both intracellular and extracellular copper ion levels, thereby shaping an immunosuppressive TME that impairs CD8⁺ T-cell function.

To further explore the relationship between cisplatin resistance and tumour immune suppression in patients with cervical cancer, peripheral blood lymphocytes were isolated from normal volunteers. CD8⁺ T cells were subsequently purified for coculture with SiHa or SiHa-DDP cells. Compared with SiHa cells, cells cocultured with SiHa-DDP cells had significantly lower levels of CD8⁺ T-cell-derived IFN-γ and GzmB, indicating that the cytotoxic activity and effector activity of CD8 + T cells were impaired (Fig. 2F–I). Proliferation and apoptosis assays demonstrated that coculture with SiHa-DDP cells markedly inhibited CD8⁺ T-cell proliferation while promoting apoptosis (Fig. 2J, K), indicating a stronger immunosuppressive capacity of cisplatin-resistant cervical cancer cells.

### ATP7A modulates the suppression of CD8^+^ T cell function

**Fig. 3:**
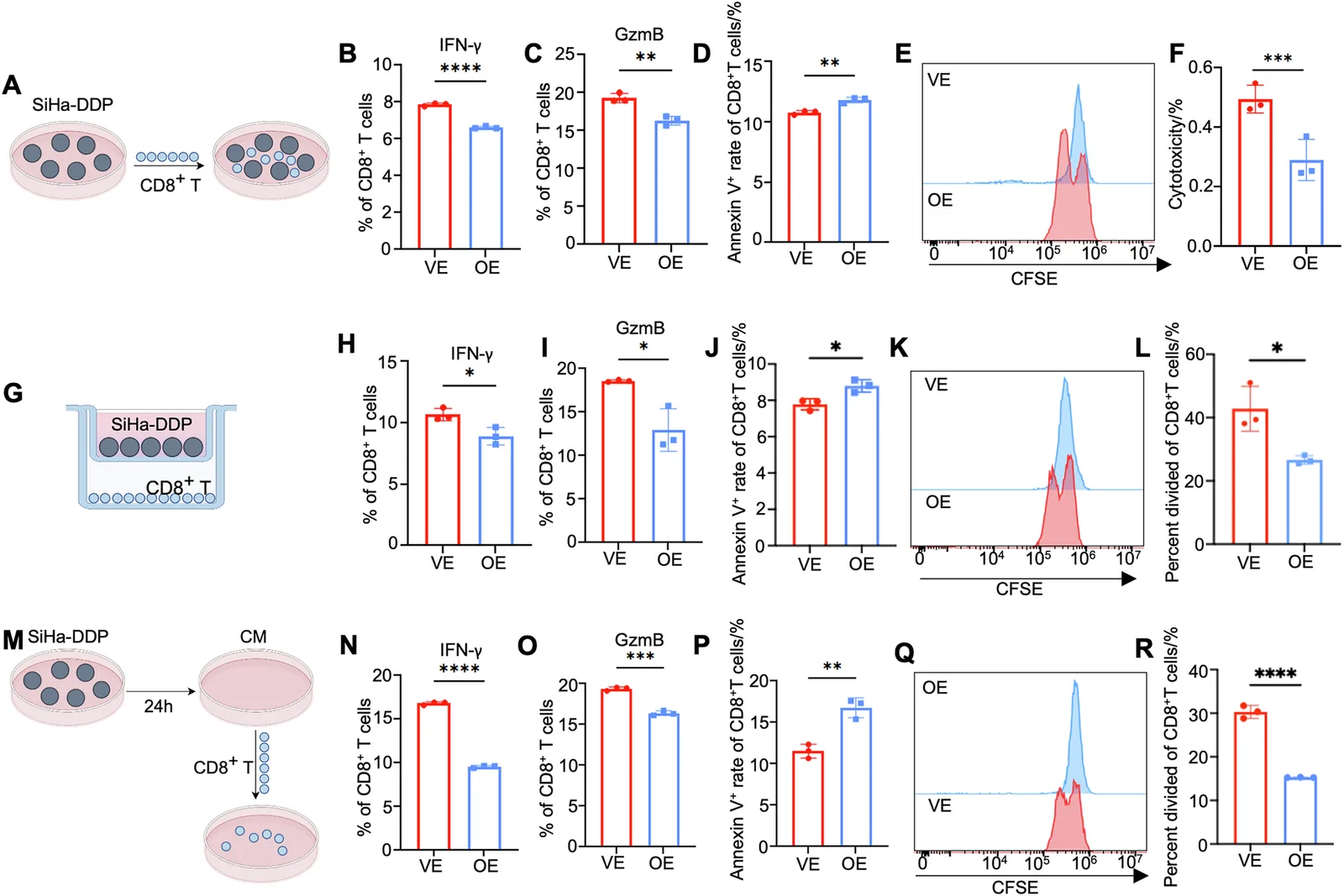
Effects of ATP7A modulation on CD8^+^ T-cell function in cisplatin-resistant cervical cancer cells. **A** Direct coculture system involving human CD8⁺ T cells and SiHa-DDP cells with ATP7A overexpression. **B**-**D** Flow cytometry analysis of IFN-γ and GzmB expression and apoptosis in CD8⁺ T cells cocultured with SiHa-DDP cells with ATP7A overexpression. **E**, **F** CFSE-based proliferation assay and cytotoxicity analysis of CD8⁺ T cells in the direct coculture system. **G** Diagram of the Transwell coculture setup used to assess the indirect effects of ATP7A on CD8⁺ T cells. **H**-**J** Flow cytometry analysis of cytokine production, proliferation, and apoptosis in CD8⁺ T cells cultured in a Transwell system with SiHa-DDP cells with ATP7A overexpression. **K**, **L** CFSE assay evaluating CD8⁺ T-cell proliferation under Transwell coculture conditions. **M** The conditioned medium (CM) culture system in which CD8⁺ T cells were treated with CM collected from SiHa-DDP cells. **N**, **P** Flow cytometry analysis of IFN-γ and GzmB expression and apoptosis in CD8⁺ T cells treated with CM from ATP7A-overexpressing SiHa-DDP cells **Q**, **R** CFSE analyses of the proliferation of CD8⁺ T cells exposed to CM.

To further investigate this mechanism, human CD8⁺ T cells were directly cultured together with SiHa-DDP cells (Fig. 3A, Fig. S5A). Flow cytometry analysis revealed that SiHa-DDP cells with ATP7A overexpression inhibited the proliferation of CD8⁺ T cells and reduced IFN-γ and GzmB production but promoted CD8⁺ T-cell apoptosis. In contrast, compared with control cells, CD8⁺ T cells cocultured with ATP7A-knockdown SiHa-DDP cells exhibited significantly increased proliferation and proportions of IFN-γ and GzmB and markedly reduced apoptosis (Fig. 3B–E, Fig. S5B–E). Furthermore, overexpression of ATP7A in SiHa-DDP cells suppressed the cytotoxic killing activity of CD8⁺ T cells (Fig. 3F, Fig. S5F). In the mouse model, CD8⁺ T cells purified from the spleens of BALB/c mice were cocultured with the murine cervical cancer cell line U14 (Fig. S4C, D). Consistent with findings from humans, ATP7A overexpression in U14 cells suppressed effector functions (IFN-γ and GzmB) and proliferation of CD8⁺ T cells, reduced cytotoxicity, and increased apoptosis (Fig. S4E–L).

To determine whether ATP7A influenced CD8⁺ T-cell function through noncontact mechanisms, an indirect Transwell-based cocultivation system was established (Fig. 3G, Fig. S5G). These results were consistent with those from direct coculture, which revealed that the overexpression of ATP7A in SiHa-DDP cells notably suppressed the proliferation of CD8⁺ T cells, cytokine release, and cytotoxic activity, whereas the corresponding parameters were reversed in CD8⁺ T cells cocultured with ATP7A-knockdown SiHa-DDP cells (Fig. 3H–L, Fig. S5H–L). Moreover, CD8⁺ T cells were cultured in conditioned medium (CM) collected from SiHa-DDP cells for 24 h (Fig. 3M, Fig. S5M). Notably, compared with the control, CM from ATP7A-overexpressing SiHa-DDP cells inhibited CD8⁺ T-cell proliferation and cytokine secretion, whereas CM from ATP7A-knockdown SiHa-DDP cells significantly promoted these cellular responses (Fig. 3N–R, Fig. S5N–R). Collectively, these findings indicated that ATP7A-mediated immunosuppression was associated with factors secreted from cisplatin-resistant cervical cancer cells.

### ATP7A suppresses CD8^+^ T cell function through the release of excess copper into the TME

Notably, the results of the CCK-8 assay revealed that under treatment with the same concentration of extracellular copper, compared with CD8⁺ T cells, cervical cancer cells exhibited significantly greater tolerance to copper stress, with SiHa-DDP cells displaying the greatest degree of copper tolerance (Fig. S4M). These findings indicated that in the high-copper tumour microenvironment, CD8⁺ T cells were more susceptible to copper-induced stress injury, whereas tumour cells, especially cisplatin-resistant cells, exhibited prominent copper stress tolerance.

**Fig. 4:**
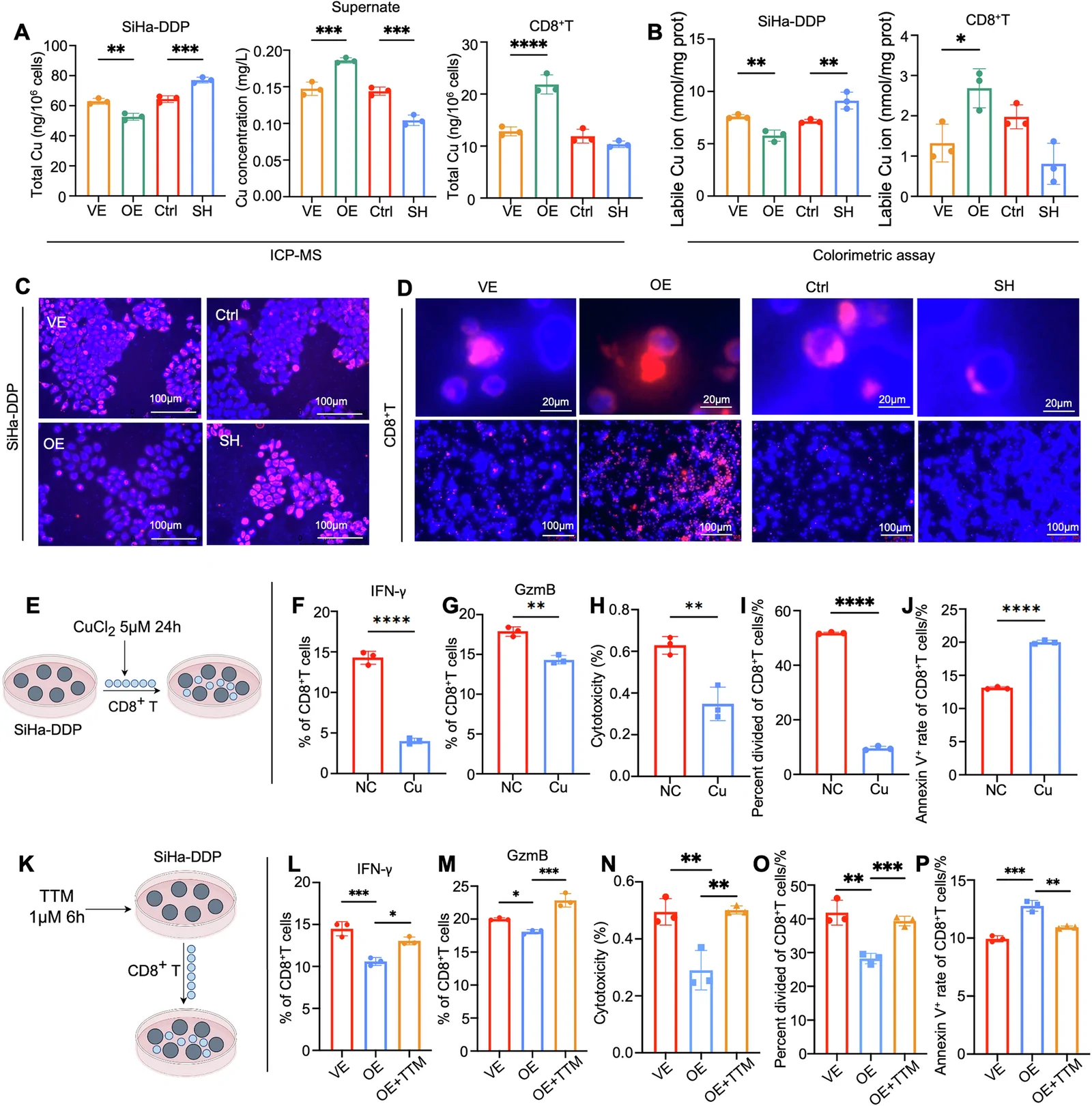
ATP7A-mediated copper release regulates CD8^+^ T-cell function. **A** ICP-MS analysis of total copper content in SiHa-DDP cells, corresponding culture supernatants and CD8⁺ T cells cocultured with SiHa-DDP cells with ATP7A overexpression or knockdown. **B** Colorimetric quantification of intracellular Cu²⁺ concentrations in co-cultured SiHa-DDP with ATP7A overexpression or knockdown and CD8⁺ T cells. **C**, **D** Fluorescence imaging of the intracellular Cu²⁺ distribution in cocultured SiHa-DDP and CD8⁺ T cells. **E** CD8⁺ T cells pretreated with 5 μM CuCl₂ for 24 h prior to coculture with SiHa-DDP cells. **F**-**J** Flow cytometry analysis of IFN-γ and GzmB expression, cytotoxic activity, and apoptosis in CD8⁺ T cells. **K** Coculture assay using SiHa-DDP cells pretreated with 1 µM TTM for 6 h. **L**-**P** Flow cytometry analysis of IFN-γ and GzmB expression, cytotoxicity, and apoptosis in CD8⁺ T cells cocultured with SiHa-DDP cells with ATP7A overexpression or TTM.

To determine whether ATP7A inhibited CD8⁺ T-cell function by promoting copper efflux into the tumour microenvironment, total copper levels (via ICP-MS) and labile copper pool levels (via colorimetric assays) were measured in the coculture system. ICP-MS and colorimetric assays consistently demonstrated that ATP7A knockdown in SiHa-DDP cells significantly increased the intracellular copper concentration in tumour cells and decreased the copper concentration in the culture supernatant. In contrast, ATP7A overexpression led to a marked increase in extracellular copper levels (Fig. 4A, B). CD8⁺ T cells exhibited the opposite pattern in the coculture system, with higher levels of accumulated intracellular copper in cells cocultured with ATP7A-overexpressing SiHa-DDP cells (Fig. 4A, B). These findings indicated that copper levels inside and outside CD8⁺ T cells were affected by copper transport in SiHa-DDP cells.

Immunofluorescence staining further validated these results. ATP7A-knockdown SiHa-DDP cells displayed stronger intracellular labile copper fluorescence signals, whereas ATP7A-overexpressing cells presented markedly weaker signals (Fig. 4C). Compared with their respective controls, CD8⁺ T cells cocultivated with tumour cells overexpressing ATP7A exhibited increased copper fluorescence, whereas those cocultured with ATP7A-knockdown tumour cells exhibited attenuated fluorescence (Fig. 4D). These results indicated that ATP7A-mediated copper export from tumour cells altered the copper ion equilibrium within the tumour microenvironment and in surrounding CD8⁺ T cells.

To further assess whether copper dysregulation directly impaired CD8⁺ T-cell function, CD8⁺ T cells were pretreated with CuCl_2_ before they were cocultured with SiHa-DDP cells (Fig. 4E). Copper-pretreated CD8⁺ T cells exhibited markedly reduced proliferation, cytotoxicity, and cytokine release (IFN-γ and GzmB) and increased apoptosis (Fig. 4F–J). Conversely, TTM pretreatment of SiHa-DDP cells alleviated ATP7A-induced immunosuppression; restored CD8⁺ T cell proliferation, cytokine production, and cytotoxicity; and reduced apoptosis (Fig. 4K–P).

Similarly, when mouse-derived CD8⁺ T cells and U14 cells were used in a murine coculture system, exogenous copper ions suppressed the function of mouse CD8⁺ T cells, whereas TTM treatment reversed the inhibitory effect on the function of mouse CD8⁺ T cells induced by ATP7A-overexpressing U14 cells (Fig. S6A–L). Collectively, these results demonstrated that ATP7A-mediated copper efflux from cisplatin-resistant cervical cancer cells disrupted copper ion homeostasis in the tumour microenvironment, resulting in impaired function of CD8⁺ T cells.

### ATP7A suppresses the activation of CD8 ^+^ T cells through inhibition of the TCR signalling pathway

**Fig. 5:**
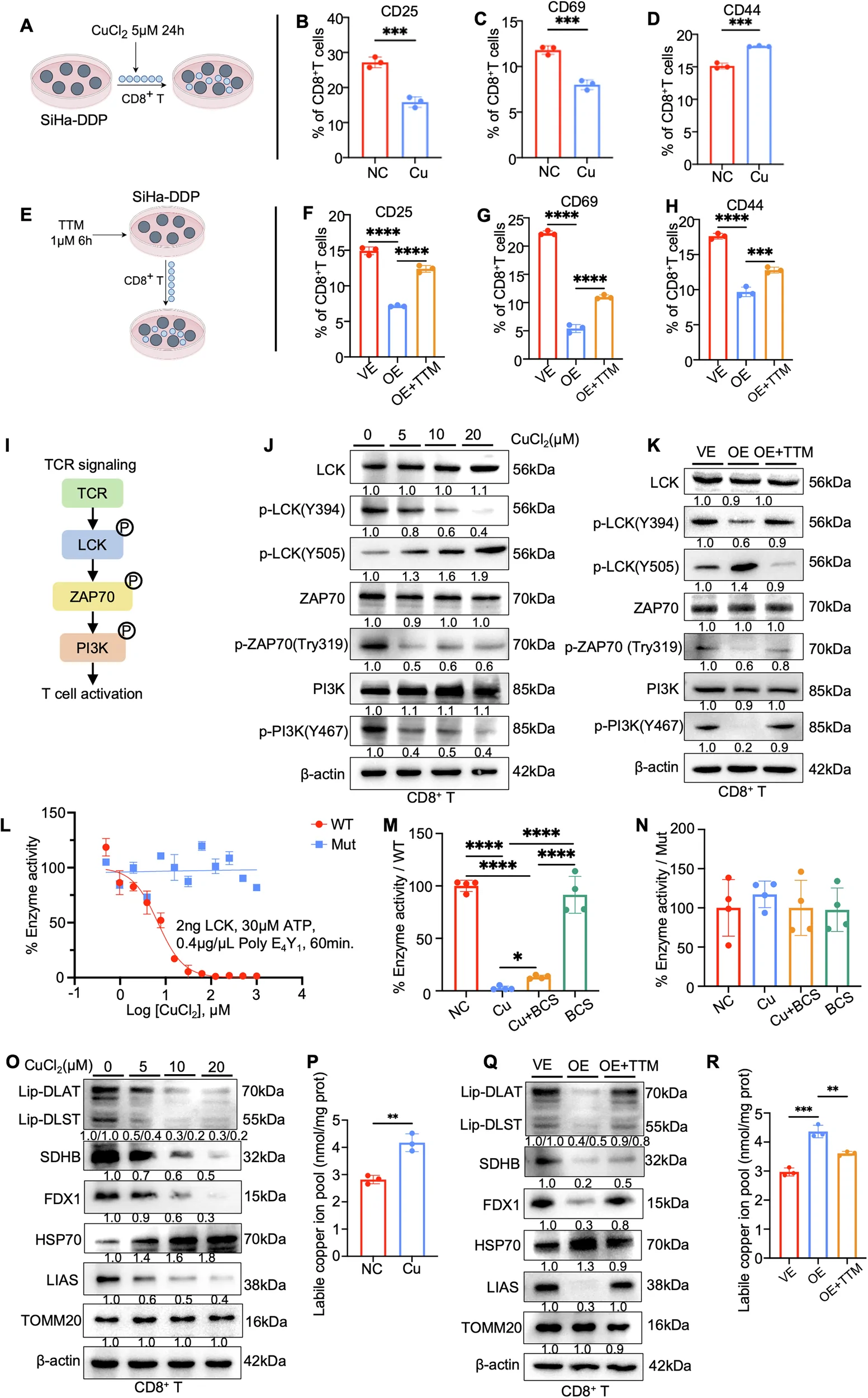
ATP7A suppresses CD8^+^ T-cell activation via copper-dependent inhibition of TCR signalling. **A** CD8⁺ T cells were pretreated with 5 μM CuCl₂ for 24 h prior to coculture with SiHa-DDP cells. **B**-**D** Flow cytometric analysis of the proportions of CD25-, CD69-, and CD44-positive CD8⁺ T cells. **E** Coculture assay using SiHa-DDP cells pretreated with 1 µM TTM for 6 h. **F**-**H** Representative flow cytometry plots and quantitative analysis of the proportions of CD25-, CD69-, and CD44-positive CD8⁺ T cells cocultured with SiHa-DDP cells with ATP7A overexpression or TTM. **I** Schematic diagram illustrating the TCR-LCK-ZAP70-PI3K signalling cascade in CD8⁺ T cells. **J** Western blot analysis of the expression of TCR signalling proteins (LCK, p-LCK (Y394), p-LCK (Y505), ZAP70, p-ZAP70 (Try319), PI3K, and p-PI3K (Y467)) in CD8⁺ T cells treated with graded concentrations of CuCl₂. **K** Western blot analysis of the expression of TCR signalling proteins in CD8⁺ T cells cocultured with SiHa-DDP cells with ATP7A overexpression or TTM. **L** Dose-response curves of Cu²⁺ on WT and Tyr394 mutant LCK kinase activity. **M**, **N** Reversal of Cu²⁺-induced inhibition by BCS. **O** Western blot and quantitative analyses of the expression of cuproptosis-related proteins (Lip-DLAT, Lip-DLST, SDHB, FDX1, HSP70, TOMM20, and LIAS) in CD8⁺ T cells treated with graded concentrations of CuCl₂. **P** Intracellular Cu^2+^ levels in CD8⁺ T cells treated with CuCl₂ (10 µM). **Q**, **R** Western blot and quantitative analyses of the levels of cuproptosis-related proteins (Lip-DLAT, Lip-DLST, SDHB, FDX1, HSP70, TOMM20, and LIAS) and intracellular Cu^2+^ in CD8⁺ T cells cocultured with ATP7A overexpression SiHa-DDP cells with TTM or not.

Since the effector activities of CD8⁺ T cells (IFN-γ and GzmB production) rely on proper activation [19], the effects of ATP7A and copper ions on this activation process were further examined. Compared with the corresponding control treatment, treatment with CuCl_2_ significantly reduced the expression of the activation markers CD25, CD69, and CD44 (Fig. 5A–D). Consistently, compared with the corresponding control group, coculture with ATP7A-overexpressed SiHa-DDP cells markedly reduced CD8 ⁺T-cell activation, as shown by decreased CD25, CD69, and CD44 expression. Importantly, pretreatment with TTM successfully abrogated the inhibitory effect of ATP7A-overexpressing SiHa-DDP cells on CD8⁺ T-cell activation (Fig. 5E–H).

The T-cell receptor (TCR) signalling pathway, which involves key mediators such as LCK, ZAP70, and PI3K that regulate downstream transcriptional activation, is essential for the activation and cytotoxic function of CD8⁺ T cells [19] (Fig. 5I). To elucidate how ATP7A and copper ions suppressed the activation of CD8^+^ T cells, graded concentrations of CuCl_2_ were applied to CD8⁺ T cells. Upon copper exposure, the total protein levels of LCK, ZAP70, and PI3K in CD8⁺ T cells remained unchanged, whereas the protein expression of p-LCK (Y394), p-ZAP70 (Try319) and p-PI3K (Y467) was markedly reduced, and the inhibitory phosphorylated form of p-LCK (Y505) was significantly upregulated (Fig. 5J). Similarly, CD8⁺ T cells indirectly cocultured with ATP7A-overexpressing SiHa-DDP cells exhibited comparable suppression of p-LCK (Y394), p-ZAP70 (Try319), and p-PI3K (Y467) protein expression, along with an increase in the level of the inhibitory phosphorylated form p-LCK (Y505), whereas pretreatment with TTM effectively alleviated these inhibitory effects (Fig. 5K). These results indicated that ATP7A-mediated copper efflux impaired CD8⁺ T-cell activation and function via the inhibition of TCR signalling.

A 50 ns molecular dynamics simulation was performed for the Cu²⁺-LCK complex to further investigate the structural mechanism underlying copper-mediated LCK inhibition. The simulation showed that Cu²⁺ stably coordinated near the activation loop residue Tyr394, perturbing its phosphorylation site conformation (Fig. S7A). Root mean square deviation (RMSD) analysis revealed greater structural fluctuations in the Cu²⁺-bound system than in the apo state (0.35–0.4 nm vs. 0.2 nm), which indicated reduced structural stability upon copper binding (Fig. S7B). Moreover, the distance between Cu²⁺ and Tyr394 remained ~2.0 Å throughout the simulation, suggesting persistent electrostatic interactions (Fig. S7C). The kinase activity assay results showed that Cu²⁺ suppressed WT-LCK activity in a dose-dependent way. By contrast, the Tyr394 mutant showed remained unresponsive to Cu²⁺, with no notable activity reduction across all tested concentrations (Fig. 5L). To assess the specificity of this inhibition, the Cu-selective chelator bathocuproinedisulfonic acid disodium salt (BCS) was applied. BCS treatment largely reversed the Cu²⁺-induced suppression of WT-LCK, bringing activity back to near-control levels, while BCS alone had no effect on kinase activity. As anticipated, no such reversal occurred in the Tyr394 mutant, consistent with its minimal basal activity and lack of Cu²⁺ sensitivity (Fig. 5M, N). Collectively, these findings suggested that Cu²⁺ directly bound to Tyr394, altering the local conformational stability of LCK and thereby inhibiting its activation at the molecular level.

### ATP7A-mediated copper efflux into the TME triggers CD8+ T cell death with cuproptosis characteristics

In addition to TCR signalling suppression, excessive copper accumulation in CD8⁺ T cells also induced cell death that exhibits key characteristics consistent with cuproptosis. CuCl_2_ treatment led to decreased expression of cuproptosis-related proteins (Lip-DLST, Lip-DLAT, FDX1, LIAS, and SDHB) and increased expression of HSP70 in CD8⁺ T cells in a dose-dependent manner, while TOMM20 protein expression showed no obvious change (Fig. 5O). Consistently, intracellular divalent copper ion levels were significantly elevated following CuCl₂ treatment (Fig. 5P). CD8⁺ T cells cocultivated with SiHa-DDP cells overexpressing ATP7A exhibited a similar reduction in the expression of cuproptosis-related proteins and a marked increase in the accumulation of intracellular copper, both of which were reversed by TTM treatment (Fig. 5Q, R).

### SOX21 transcriptionally regulates ATP7A to promote cisplatin resistance and immune suppression

**Fig. 6:**
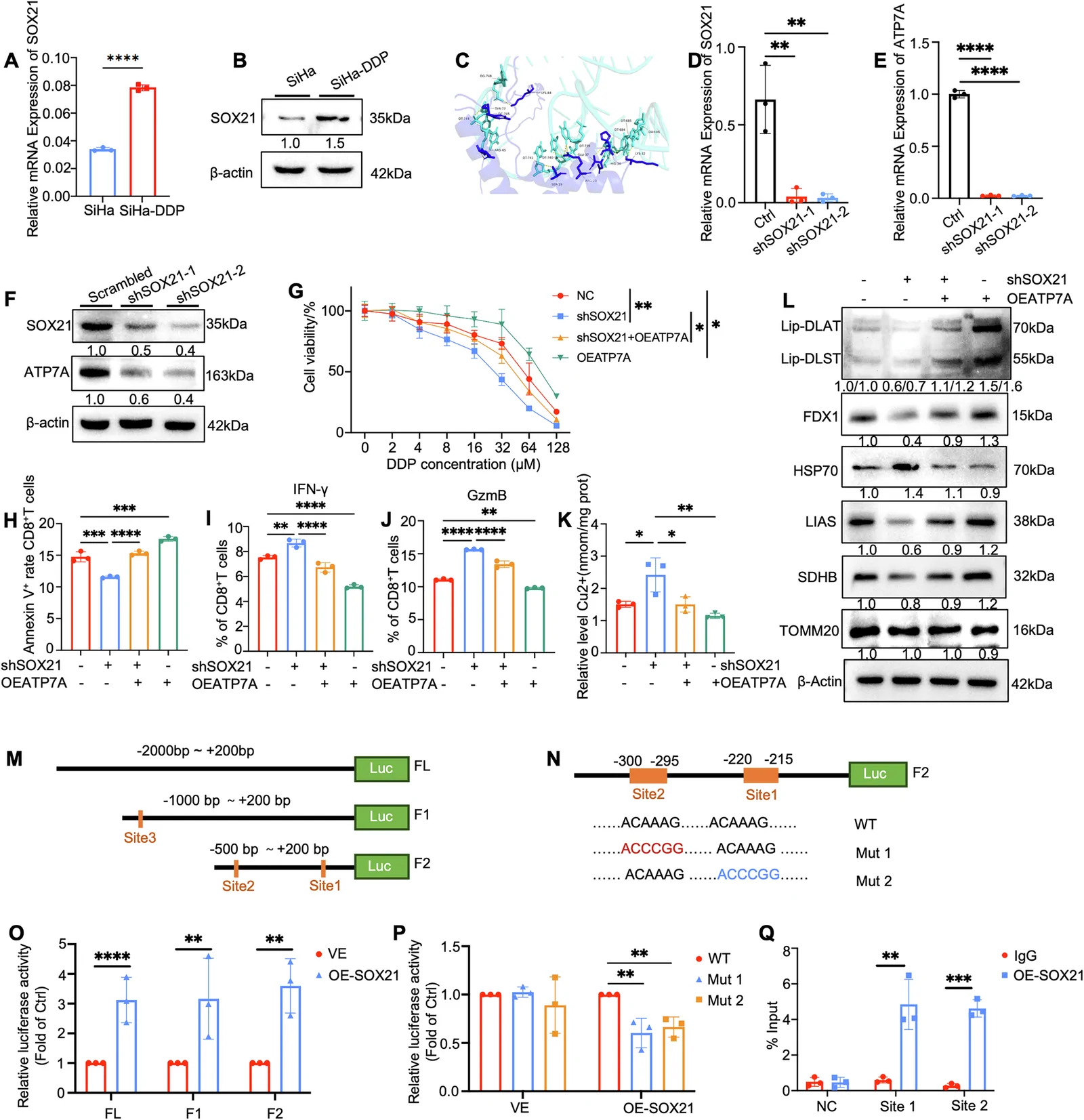
SOX21 regulates ATP7A transcription and contributes to cisplatin resistance and immune suppression in cervical cancer. **A**, **B** qRT-PCR and Western blot analyses of SOX21 mRNA and protein levels in SiHa and SiHa-DDP cells. **C** Predicted molecular docking model illustrating the binding of SOX21 to the ATP7A promoter. **D**-**F** qRT-PCR and Western blot analysis of SOX21 and ATP7A expression after SOX21 knockdown. **G** Cisplatin sensitivity in SiHa-DDP cells after SOX21 knockdown or ATP7A overexpression. Flow cytometry of CD8⁺ T cells cocultured with SiHa-DDP cells after SOX21 knockdown or ATP7A overexpression to assess apoptosis (**H**), IFN-γ (**I**), GzmB (**J**), and copper levels (**K**). **L** Western blot analysis of the expression of cuproptosis-related proteins (FDX1, LIAS, SDHB, HSP70, TOMM20, Lip-DLAT, and Lip-DLST) in SiHa-DDP cells after SOX21 knockdown or ATP7A overexpression. **M** Schematic diagram of ATP7A promoter luciferase reporter constructs covering FL, F1 and F2. **N** Predicted SOX21-binding motifs within the F2 region. **O**, **P** Dual-luciferase reporter assays assessing ATP7A promoter activity in SiHa-DDP cells using constructs containing full-length (FL), F1, and F2 fragments and mutant variants (Mut1 and Mut2). **Q** ChIP-qPCR assay examining SOX21 enrichment at the ATP7A promoter region in SiHa-DDP cells, with the NC region serving as a negative control.

Transcription factors associated with ATP7A expression (correlation coefficient ≥ 0.65) were identified using the TF-Target-Finder platform. Candidates were then prioritized based on their association with cisplatin resistance. SOX21 showed the strongest and most consistent correlation with both ATP7A expression and cisplatin sensitivity and was therefore selected for further analysis (Fig. S7D). Accordingly, the role and underlying mechanisms of SOX21 in cisplatin resistance and immune suppression in cervical cancer were further investigated. Survival analysis revealed that high SOX21 expression was associated with poor prognosis among cancer patients (Fig. S7E). Both the protein and mRNA levels of SOX21 were more elevated in SiHa-DDP cells than in parental SiHa cells (Fig. 6A, B). Molecular docking analysis further revealed a direct binding interaction between SOX21 and the ATP7A promoter sequence (Fig. 6C). SOX21 knockdown reduced ATP7A protein and mRNA levels in SiHa-DDP cells (Fig. 6D–F). Furthermore, SOX21 knockdown increased the susceptibility of SiHa-DDP cells to cisplatin, whereas ATP7A overexpression reversed this effect (Fig. 6G). Moreover, CD8⁺ T cells cocultured with SOX21-knockdown SiHa-DDP cells exhibited decreased apoptosis and increased IFN-γ and GzmB secretion, which were reversed by ATP7A overexpression (Fig. 6H–J). In addition, SOX21 knockdown triggered cuproptosis in SiHa-DDP cells, as indicated by increased intracellular copper levels and dysregulated expression of cuproptosis-related proteins, and these effects were reversed by ATP7A overexpression (Fig. 6K, L). These findings suggested that SOX21 promoted cisplatin resistance and immune suppression in cervical cancer by upregulating ATP7A expression.

To explore the transcriptional regulatory mechanism, three probable SOX21 binding sites were detected within the ATP7A promoter region through analysis of the JASPAR database (-2000 to +200 bp): two high-confidence sites (Site 1 and Site 2, -220 bp and −295 bp) and one low-confidence site (Site 3, -1000 bp) (Fig. 6M, N). To functionally validate these predictions, luciferase reporter plasmids containing different promoter fragments (FL, F1, and F2) in SiHa-DDP cells were constructed. Dual-luciferase reporter assays revealed that cotransfection with SOX21 significantly increased reporter activity across all the constructs (Fig. 6O). Consistently, knockdown of SOX21 markedly attenuated the luciferase activity of the ATP7A promoter reporter (Fig. S7F), which indicated that SOX21 activated ATP7A promoter transcription in the context of DDP resistance.

Site-directed mutagenesis was subsequently performed on the F2 construct to disrupt Site 1 (Mut1) and Site 2 (Mut2). The results revealed that SOX21 robustly activated transcription from the wild-type F2 construct, while mutation of either binding site markedly weakened this transcriptional impact (Fig. 6P, Fig. S7G). Consistently, ChIP-qPCR confirmed the significant enrichment of SOX21 binding at Site 1 and Site 2 in SiHa-DDP cells (Fig. 6Q, Fig. S7H). Collectively, these results demonstrated that SOX21 directly bound to the ATP7A promoter, increased its transcriptional activity, and thereby upregulated ATP7A expression, contributing to cisplatin resistance and immune suppression in cervical cancer.

### ATP7A promotes immune suppression and cisplatin resistance in vivo

**Fig. 7:**
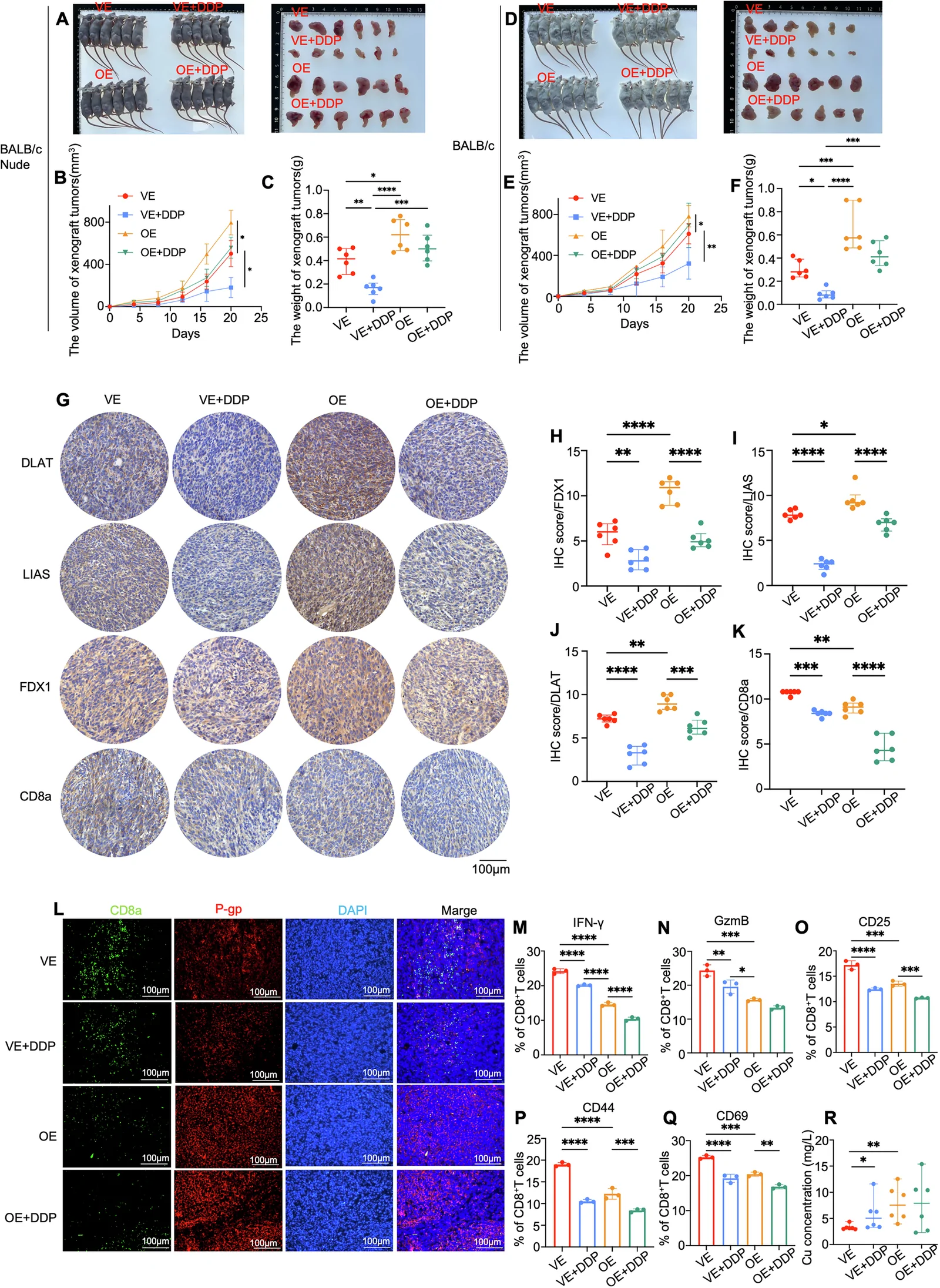
ATP7A overexpression promotes cisplatin resistance and modulates tumour immunity in vivo. Four experimental groups were established: VE (control), OE (ATP7A overexpression), VE + DDP (cisplatin), and OE + DDP (ATP7A overexpression+cisplatin). **A**-**C** Representative images and growth curves of subcutaneous xenograft tumours in BALB/nude mice from each experimental group. **D**-**F** Tumour formation and growth in immunocompetent BALB/c mice from each experimental group. **G**-**K** IHC staining of tumour sections for DLAT, LIAS, FDX1, and CD8a proteins and quantification of IHC scores for each experimental group. **L** Multiplex immunofluorescence images showing CD8a (green), P-gp (red) and DAPI (blue) staining in tumour tissues from each experimental group. **M**-**Q** Flow cytometric analysis of tumour-infiltrating CD8⁺ T cells to measure IFN-γ, GzmB, CD25, CD44, and CD69 expression in each experimental group. **R** ICP–MS analysis of copper concentrations in the TIFs from each experimental group.

In subcutaneous xenograft cervical cancer models using nude mice, cisplatin administration notably inhibited tumour growth, whereas ectopic expression of ATP7A significantly enhanced tumour growth. Notably, ATP7A overexpression significantly weakened the antitumour effect of cisplatin, revealing reduced cisplatin sensitivity in tumours with high ATP7A expression (Fig. 7A–C). Consistent results were observed in immunocompetent BALB/c mice, further confirming that ATP7A overexpression conferred cisplatin resistance in vivo (Fig. 7D–F).

Immunohistochemical analysis of subcutaneous xenograft tumours revealed that cisplatin treatment markedly increased the expression of the cuproptosis-related proteins DLAT, LIAS, and FDX1, indicating enhanced cuproptosis. In contrast, ATP7A overexpression attenuated the cisplatin-induced changes, suggesting that elevated ATP7A expression suppressed cuproptosis (Fig. 7G–J). Furthermore, compared with the control treatment, both ATP7A overexpression treatment and cisplatin treatment markedly reduced CD8⁺ T-cell infiltration in tumour tissues (Fig. 7K). Multicolour immunofluorescence further revealed elevated P-gp expression and decreased CD8⁺ T-cell density in ATP7A-overexpressing tumours, with a negative correlation between P-gp levels and CD8⁺ T-cell abundance (Fig. 7L). These findings demonstrated a close link between cisplatin resistance, immune suppression, and ATP7A expression in tumour cells.

To further evaluate the effect of ATP7A on the function of CD8⁺ T cells, the expression of activation and effector markers was analysed in CD8^+^ T cells infiltrating tumours (Fig. S7I). ATP7A-overexpressing tumours exhibited significantly reduced proportions of IFN-γ-, GzmB-, CD25-, CD69-, and CD44-positive CD8⁺ T cells, indicating impaired activation and cytotoxicity (Fig. 7M–Q). Additionally, ICP-MS revealed an increase in copper ion levels in the tumour interstitial fluid of ATP7A-overexpressing tumours (Fig. 7R).

## Discussion

Copper homeostasis has evolved into a crucial factor in tumorigenesis [20]. In this study, large-scale population data from the NHANES 2011–2016 cohort revealed that elevated serum copper levels were significantly correlated with increased pan-cancer mortality. Moreover, compared with those in healthy controls, copper ion concentrations in both the blood and tumour tissues of patients with cervical cancer were markedly elevated. At the tissue level, both cervical cancer specimens and matched patient sera exhibited markedly higher copper concentrations than did normal controls. These findings aligned with previous epidemiological reports linking systemic copper overload to oncogenic signalling, oxidative stress and mitochondrial dysfunction [21–23]. Thus, these data indicate that copper accumulation is a distinctive feature of cervical cancer pathogenesis.

Cells maintain stable copper levels via a precise transport system, which mainly relies on two key types of transporters to control copper flow direction. High‑affinity uptake proteins including CTR1/SLC31A1 and CTR2 carry external copper into cells [10]. In contrast, P‑type pump proteins such as ATP7A and ATP7B remove excess copper from cells or store it inside organelles [24]. In this study, ATP7A was identified as the most abundantly expressed copper transporter in cervical cancer cells. The protein expression of ATP7A was greater in cisplatin-resistant cervical cancer cells than in cisplatin-sensitive cells and was related to poor prognosis in patients with cervical cancer. Furthermore, this is the first study to show that ATP7A overexpression significantly enhanced cisplatin resistance, whereas ATP7A inhibition activated cuproptosis. As a result, ATP7A-mediated copper efflux protected tumour cells from cuproptosis and facilitated the development of drug resistance in cervical cancer.

Cisplatin resistance arises not only from tumour-intrinsic genetic or metabolic alterations but also from the establishment of an immunosuppressive tumour microenvironment. Coculture experiments in this study revealed that SiHa-DDP cells markedly suppressed CD8⁺ T-cell function, including the reduction of cell proliferation, cytotoxicity, and cytokine secretion (IFN-γ and GzmB). Previous studies have reported similar findings, showing that drug-resistant tumour cells induced CD8⁺ T-cell exhaustion and apoptosis through the secretion of immune-inhibitory cytokines such as IL-10 and TGF-β or through the release of exosome-mediated signals such as galectin-9, PD-L1, and arginase-1 (ARG1) [25, 26]. TCR signaling acts as a central regulator coordinating effector function and survival of CD8⁺ T cells. Its attenuation not only suppresses IFN-γ and GzmB expression but also predisposes T cells to apoptosis through impaired survival signaling and exhaustion-associated pathways [27]. These processes weaken immune clearance, leading to chemoresistance and immune suppression by tumour cells.

As a key copper efflux transporter, ATP7A profoundly influences intracellular and extracellular copper homeostasis when it is highly expressed in tumour cells. In this study, ATP7A overexpression or excess copper suppressed the activation of CD8⁺ T cells in coculture systems. These findings revealed a mechanistic link between ATP7A-mediated copper export and impaired antitumour immunity. Consistent with our findings, earlier research revealed a link between the expression of ATP7A and the proportion of CD8⁺ T cells [28]. In addition, Li et al. [29] reported that copper accumulation impaired CD8^+^ T cell mitochondrial respiration and energy metabolism, thereby weakening immune function. Excessive copper has been demonstrated to trigger ROS production and oxidative stress and further impair CD8⁺ T-cell activation [30]. Interestingly, this study revealed that the immunosuppressive phenotype exhibited by drug-resistant cells was correlated with elevated ATP7A levels and enhanced tolerance to copper stress. These findings suggested that dysregulated copper metabolism served as a metabolic checkpoint connecting chemoresistance with immune evasion by suppressing the activation and effector function of CD8⁺ T cells.

CD8⁺ T cells exhibit a metabolic landscape distinct from tumor cells. Activated CD8⁺ T cells predominantly rely on glycolysis for energy supply, whereas tumor cells frequently display enhanced mitochondrial metabolism, particularly increased oxidative phosphorylation and TCA cycle activity, metabolic features closely associated with cuproptosis sensitivity [31]. This study confirms that excess copper induces CD8⁺ T cell death accompanied by abnormal lipoylated protein expression and mitochondrial dysfunction. Nevertheless, the concurrent involvement of other cell death pathways cannot be fully excluded at this stage. Although our findings suggest that excessive copper may contribute to CD8⁺ T-cell dysfunction, the involvement of cuproptosis should be interpreted with caution. Cuproptosis was originally characterized in metabolically active tumor cells with high dependence on mitochondrial respiration, whereas CD8⁺ T cells exhibit distinct metabolic features that may influence their susceptibility to this form of cell death. Therefore, the observed effects of copper accumulation in CD8⁺ T cells may not be exclusively attributable to classical cuproptosis and could involve other forms of cell death or functional impairment. Further studies are needed to delineate the specific mechanisms underlying copper-induced cytotoxicity in CD8⁺ T cells.

It has been reported that copper modulates the phosphorylation and activation of multiple kinases [32–34]. Notably, copper promotes MEK1/2 activation and sustains oncogenic BRAF signalling, whereas copper depletion or binding site mutation diminishes ERK phosphorylation and tumour progression [34]. At the mechanistic level, ATP7A overexpression or Cu²⁺ exposure inhibited TCR signalling, likely through Cu²⁺ coordination near Tyr394 within the LCK activation loop. Excessive amounts of copper induced conformational changes in LCK, thereby hindering its activation and downstream phosphorylation. Collectively, these findings suggested that copper might similarly regulate LCK activity within the TCR signalling pathway, analogous to its role in the MEK1/2-BRAF axis.

SOX21 plays important roles in tumour initiation and progression and is a transcription factor belonging to the SRY-related HMG-box family [35]. Previous studies have shown that other SOX family members, such as SOX2 and SOX9, promote drug resistance, metabolic reprogramming, and immune evasion in various cancers [36–38]. However, the role of SOX21 in cisplatin resistance and immune suppression has not been investigated. In this study, SOX21 was first shown to be markedly upregulated at both the protein and mRNA levels in cisplatin-resistant cervical cancer cells. Functional assays demonstrated that SOX21 silencing inhibited ATP7A expression, enhanced cisplatin sensitivity, and restored CD8⁺ T-cell cytotoxicity, whereas ATP7A overexpression reversed these effects. Dual-luciferase and ChIP–qPCR assays confirmed that SOX21 directly bound to the ATP7A promoter to activate its transcription. Therefore, these results provide new insight into therapeutic strategies targeting the SOX21-ATP7A-copper pathway to restore antitumour immunity and mitigate cisplatin resistance.

A limitation of this study is that the mechanism of copper influx in CD8⁺ T cells was not directly investigated, with metal ion homeostasis inferred only from transcriptional profiles. Although our findings, together with bioinformatic analyses and previous studies, suggest a potential role for SLC31A1 (CTR1) in copper uptake under conditions of increased extracellular copper, the precise regulatory mechanisms remain to be fully elucidated. Further studies using genetic or pharmacological approaches targeting copper transporters are needed to clarify this issue. In addition, due to the limited availability of primary human CD8⁺ T cells, comprehensive protein-level validation of TCR signaling could not be performed in human samples. Therefore, most mechanistic experiments were conducted in mouse CD8⁺ T cells, which may not fully recapitulate human immune responses.

**Fig. 8:**
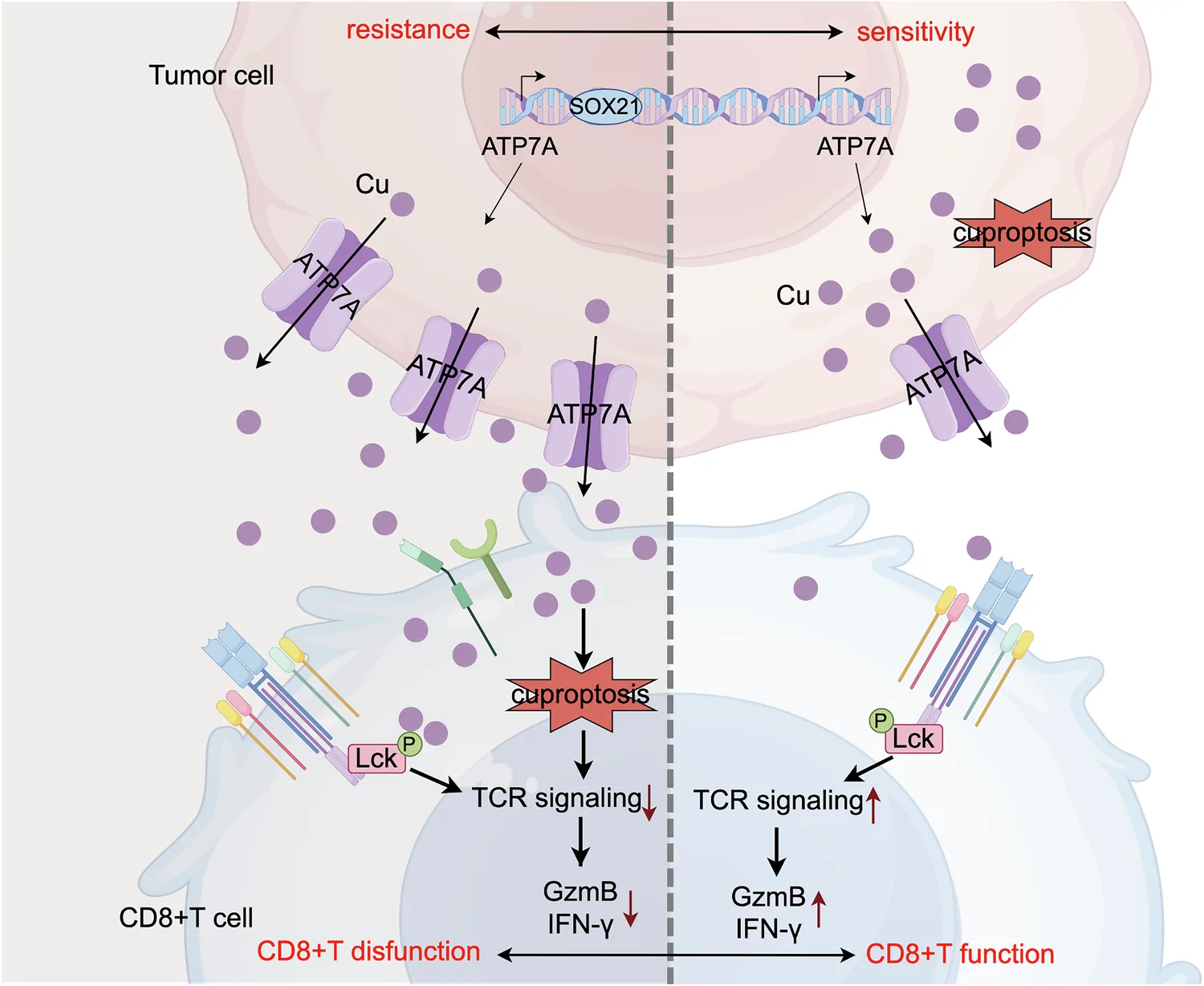
Schematic illustration of the proposed SOX21-ATP7A-copper regulatory axis in cisplatin-resistant cervical cancer. By Figdraw (www.figdraw.com, copyright authorization ID: ASPYS1190c).

In conclusion, this study reveals a previously unrecognized regulatory axis linking copper metabolism to cisplatin resistance and immune suppression in cervical cancer. Elevated copper levels contribute to an immunosuppressive tumour microenvironment, in which ATP7A acts as a central mediator driving both chemoresistance and CD8⁺ T-cell dysfunction. Furthermore, ATP7A-driven copper efflux inhibits LCK activation and disrupts TCR signalling, leading to impaired CD8⁺ T-cell cytotoxicity. In addition, SOX21 transcriptionally upregulates ATP7A, forming a SOX21-ATP7A-copper axis that enhances tumour survival under cisplatin stress. Collectively, these findings establish copper dysregulation as a critical bridge between metabolic reprogramming and immune escape, highlighting the therapeutic potential of targeting ATP7A or the copper pathway in cervical cancer to restore cisplatin sensitivity and reinvigorate antitumour immunity (Fig. 8).

## Materials and methods

### Bioinformatics mining

Data from the 2011–2016 wave of NHANES data (https://www.cdc.gov/nchs/nhanes/index.html) were analysed. Participants with a self-reported history of cancer (MCQ220 = 1) were included, and those for whom serum copper concentration, survival time, or vital status were missing were excluded. Serum copper levels were dichotomized into high and low groups using the best cut-off value identified via the maximum selected rank test (surv cut-off point, minprop=0.10). Kaplan–Meier curves with log-rank tests revealed differences in survival between the two groups.

Clinical data alongside RNA-seq data of cervical cancer (TCGA-CESC) were retrieved from the TCGA, and scRNA-seq data (GSE208653 and GSE197461) were processed with Seurat (v4.3.0) in R. After quality control, cells were clustered by t-SNE and annotated into major immune and stromal types on the basis of canonical markers. Copper-related activity was calculated using AUCell. Correlations among gene expression levels were analysed using Spearman’s correlation. Correlations between gene expression and immune infiltration scores (GSE554) were evaluated using ssGSEA. To predict cisplatin resistance, Elastic Net and XGBoost models with fivefold cross-validation were constructed. Patients in the TCGA-CESC cohort were grouped into cisplatin-resistant and cisplatin-sensitive groups, and model performance was evaluated by the area under the curve (AUC) and receiver operating characteristic (ROC) curves. The prognostic impact of gene expression was determined by Kaplan–Meier survival analysis. To explore potential upstream regulators, transcription factor prediction and binding site analysis were performed using the Harmonizome (https://jingege.shinyapps.io/TF_predict/) and JASPAR (https://jaspar.elixir.no/) databases. Bioinformatics and machine learning analyses were implemented in R using the following packages: Seurat, AUCell, survival, glmnet, xgboost, limma, clusterProfiler, survminer, ggplot2, and ComplexHeatmap. The custom code supporting this study is available from the corresponding author on reasonable request.

### Cell culture and experimental treatment

SiHa, HeLa, C33A, CaSki, MS751, and U14 cell lines were acquired from the American Type Culture Collection (ATCC, USA) and maintained in RPMI-1640 medium (Meilunbio, China) or Dulbecco’s modified Eagle’s medium (DMEM; Meilunbio, China) supplemented with 10% foetal bovine serum (SunRise Biotechnology, China) and 1% penicillin–streptomycin (Meiluncell, China). Cisplatin-resistant SiHa-DDP sublines were generated from wild-type SiHa cells through stepwise treatment (0.5, 1, 2, 4, 6, and 10 μM) with increasing concentrations of cisplatin for 6–9 months. All the cells were cultured in a humidified incubator with 5% CO₂ at 37 °C.

For in vitro experiments, copper chloride (CuCl₂, MCE, HY-B0527, USA) was applied at final doses of 0, 5, 10, and 20 μM over a 24 h period to investigate the effects of copper on cancer cells and CD8⁺ T cells. The copper chelator tetrathiomolybdate (TTM, MCE, HY-100, USA) was used at 1 μM for 6 h to chelate intracellular copper and inhibit cuproptosis. Cisplatin (DDP, MCE, HY-17394, USA) was administered at concentrations of 0, 2, 4, 8, 16, 32, 64, and 128 μM for 48 h to assess cytotoxicity and drug sensitivity.

### Cell transfection and virus infection

All cDNA constructs for overexpression (OE-ATP7A and OE-SOX21) and shRNA constructs for gene knockdown (shATP7A and shSOX21) were constructed by Youbio Biosciences, Inc. (Changsha, China). The cDNA sequences of ATP7A and SOX21 were inserted into the pcDNA3.1–3×Flag-C vector or pLVX-shRNA-Puro vector. Plasmid transfection was executed using Lipofectamine (Meilunbio, MA0672, China) at a 1:1000 ratio according to the manufacturer’s instructions. Plasmid DNA was blended with Opti-MEM serum-free medium (Gibco, USA) to generate DNA-Lipofectamine complexes, which were then incubated for 15 min at ambient temperature before being added to the target cells. Following 48 h of cultivation, the lentivirus-containing supernatants were collected and used to infect the target cells. After puromycin (MCE; HY-B1743, China) selection, the transfection efficiency was confirmed by western blot analysis.

### Isolation and purification of CD8^+^ T cells

Human CD8⁺ T cells were isolated from the peripheral blood of healthy volunteers, with approval from the Medical Ethics Committee of the Second Affiliated Hospital of Wenzhou Medical University (2025-K-272-01). Primary CD8⁺ T cells from human sources were extracted and purified from the peripheral blood of donors without underlying diseases. Peripheral blood mononuclear cells (PBMCs) were extracted with a PBMC extraction kit (Solarbio, P8680, China). The CD8⁺ T cells were then further purified through negative magnetic selection (BioLegend, 480012, USA). CD8⁺ T cells were cultured in RPMI-1640 medium (Meilunbio, China) supplemented with 10% FBS (PAN-Biotech, Germany) and 1% penicillin–streptomycin (Meiluncell, China), and the concentration of recombinant human interleukin-2 (IL-2) in the cell suspension was adjusted to 10 ng/mL (PeproTech, 212-12/200-02, USA). Murine CD8⁺ T cells were isolated directly from freshly harvested BALB/c mouse spleens. Gentle dissociation of the spleens was performed using a 70 μm cell strainer, with subsequent red blood cell (RBC) depletion using RBC lysis buffer (BioLegend, Cat. No. 420301, USA). Afterwards, negative magnetic selection (BioLegend, 480054, USA) was employed to obtain purified murine CD8⁺ T cells following the manufacturer’s recommended procedures. Then, anti-CD3 (Thermo, 16-0031-82, USA) and anti-CD28 (Thermo, 16-0281-82, USA) antibodies at 2 μg/mL each were used to activate the murine CD8^+^ T cells, whereas human peripheral CD8⁺ T cells were stimulated using anti-CD3/CD28 Dynabeads at a ratio of 1:1 for 48 h (Tongli Haiyuan, TL-624KIT, China).

### Cell viability detection

A cell counting kit-8 (CCK-8; Meilunbio, China) was used to evaluate cell viability. Cells were plated in 96-well plates and treated as indicated. Ten microlitres of CCK-8 reagent was added to 100 µL of fresh culture medium in each well, and the mixture was incubated at 37 °C for 2 h following treatment. A microplate reader was used to quantify the absorbance at 450 nm. Dose–response curves and half-maximal inhibitory dose (IC₅₀) values were calculated using nonlinear regression modelling in GraphPad Prism 9.

### Colony formation

Cells were seeded into six-well plates at an appropriate density and cultured in DMEM supplemented with a 10% insulin-serum mixture for two weeks. The colonies were then fixed with 4% paraformaldehyde for 30 min at room temperature and stained with 0.1% crystal violet solution for 10 min to visualize colony formation.

### Cytotoxicity assays

After coculture, the cell-free supernatants were collected, and the level of cytotoxicity was measured using a lactate dehydrogenase (LDH) cytotoxicity assay kit (Beyotime, C0017, China). Briefly, each well was loaded with 60 μL of freshly prepared LDH reaction mixture, followed by incubation for 30 min at 37 °C in the dark. A microplate reader was used to measure the absorbance at 490 nm, with 600 nm as the reference wavelength (Bio-Rad, USA). The percent cytotoxicity was computed using the following formula: Cytotoxicity (%) = (ODsample-ODcontrol)/(ODmax-ODcontrol) × 100.

### Western blot

Radioimmunoprecipitation assay (RIPA) buffer containing phenylmethylsulphonyl fluoride (PMSF) (Beyotime, China) was used for cell lysis. Using SDS–polyacrylamide gel electrophoresis (SDS–PAGE), equal amounts of protein (20 µg) were fractionated and subsequently transferred to PVDF (polyvinylidene difluoride) membranes. Following a 2 h blocking step at room temperature, the membranes were incubated with specific primary antibodies (including MRP1 (#97760), P-gp (#13978) and p-ZAP70 (Try319, #2717) from Cell Signaling Technology (USA); ATP7A (DF8506), GST-π (DF7462), LCK (AF6101), p-LCK (Tyr394, AF3101), p-LCK (Tyr505, AF3100), LIAS (PH2927), SDHB (T56860), and ZAP70 (AF6312) from Affinity Biosciences (China) and Abmart (China); PI3K (ab302958), p-PI3K (Y467, ab278545), FDX1 (ab108257), Lipoic acid (ab58724) and γ-H2AX (ab11174) from Abcam (UK); TOMM20 (#AF5206) and β-actin (#T0022) from Affinity Biosciences (China); HSP70 (T55496) from Abmart (China); and SOX21 (AF3538) from R&D Systems (USA)) overnight at 4 °C, followed by incubation with secondary antibodies conjugated to horseradish peroxidase (HRP) (Biosharp, China) for 2 h. The protein bands were visualized using an enhanced chemiluminescence (ECL) detection kit (Meilunbio, China). ImageJ software was applied to determine the relative protein expression levels.

### RNA isolation and quantitative real-time PCR

Total RNA was isolated using RNA-easy isolation reagent (Vazyme Biotech, China). The RNA amount and integrity were assessed with a NanoDrop 2000 spectrophotometer (Thermo, USA). Total RNA (1 µg) was used for cDNA synthesis with the EasyScript^®^ All-in-One First-Strand cDNA Synthesis SuperMix for qPCR (One-Step gDNA Removal, TransGen Biotech, China). qPCR was subsequently performed with TB Green Premix Ex Taq II (Takara, Japan), and GAPDH was used as an endogenous reference. All primer sequences were designed and constructed by Beijing Tsingke Biotech Co., Ltd. (China) and are listed in Supplementary Table S1.

### Copper ion detection

Cancer cells were fixed with 4% paraformaldehyde for 20 min and stained with a copper ion-selective fluorescent probe (MCE, HY-150086, China) for 20 min. CD8⁺ T cells were seeded onto L-polylysine-coated coverslips before fixation and staining under identical conditions. Nuclei were counterstained with DAPI mounting medium (Abcam, UK), and fluorescence images were captured using a confocal microscope.

The levels of intracellular Cu²⁺ were determined using a copper content assay kit (Micro Method) (Solarbio, BC5755, China). Briefly, approximately 5 × 10⁶ cells were collected, lysed by ultrasonic disruption on ice, and spun at 13,500 rpm for 10 min at 4 °C. Reagents I and II were used to mix the supernatants according to the kit protocol, and the mixture was incubated at 37 °C for 5 min. The optical density (OD) was measured at 580 nm using a microplate reader (Bio-Rad, USA). Cu²⁺ concentrations were derived from the calibration curve and expressed as nmol per 10⁶ cells or nmol per mg protein.

### In vitro kinase activity assay

WT-LCK (MCE, HY-P73269, China) and the Tyr394 mutant (tyrosine‑to‑phenylalanine, constructed by Shanghai Decode Biotechnology) were used. Kinase activity was tested using the ADP‑Glo™ kit (Promega, V6930, USA) with minor changes. The 1× kinase buffer contained SignalChem buffer I (SignalChem, K01‑09, Canada). Each 5 μl reaction in a 384‑well white plate included 0.8 ng/μl LCK (WT or Y394F), 0.4 μg/μl Poly (Glu,Tyr) 4:1 substrate (Yuanye, S29689, China), 30 μM ATP (from the kit), and different amounts of CuCl₂ or BCS. The reaction started with ATP and lasted 60 min at room temperature. Then 5 μl of ADP‑Glo™ reagent was added to stop the reaction and remove leftover ATP, followed by 40 min of incubation. Next, 10 μl of Kinase Detection Reagent was added to turn ADP into ATP and produce light through luciferase. After 30 min, luminescence was read with on a Varioskan LUX microplate reader.

### Chromatin immunoprecipitation

A chromatin immunoprecipitation (ChIP) kit (Absin, abs50034, China) was used to investigate the immediate interaction of SOX21 with the promoter region of ATP7A. The cells were fixed with 1% formaldehyde for 10 min, and the reaction was terminated by quenching with 0.125 M glycine. After the nuclei were isolated, the chromatin was sheared via sonication to generate 200–800 bp chromatin fragments. The sheared chromatin was incubated at 4 °C overnight with anti-DDDDK tag (Abcam, ab125243, UK) or normal mouse IgG (negative control) in the presence of protein A/G magnetic beads. The bound protein–DNA assemblies were recovered after extensive washing, and the crosslinks were reversed at 65 °C. DNA was subsequently isolated and quantified by qPCR using primers targeting the ATP7A promoter (Site 1 and Site 2) or a gene desert region as a negative control. After enrichment was normalized to the input DNA, the data are presented as the percentage of the input.

### Dual-luciferase reporter assay

Fragments of the ATP7A promoter (FL: −2000 ~ + 200 bp; F1: −1000 ~ + 200 bp; F2: −500 ~ + 200 bp) were subsequently cloned and inserted into the pGL4.17-luciferase reporter vector. Putative SOX21 binding sites within the F2 fragment were individually mutated (Mut 1 and Mut 2) by site-directed mutagenesis. All plasmids were constructed by Sangon Biotech (China). For luciferase reporter assays, SiHa-DDP cells were dually transfected with the indicated reporter plasmids and pcDNA3.1-SOX21 (or empty vector). As an internal control, the pRL-TK Renilla luciferase plasmid was cotransfected with target plasmids using Lipofectamine 2000 (Meilunbio, China). After 24 h, the Dual-Luciferase^®^ reporter assay system (Promega, E1910, USA) was used to measure firefly and Renilla luciferase activities in sequence on a Varioskan LUX microplate reader. Relative luciferase activity, defined as the firefly to-Renilla ratio, was normalized to the relative activity of empty vector-cotransfected cells (Ctrl).

### Animal experiments

BALB/c and BALB/c nude mice (aged 7 weeks) were purchased from Beijing Vital River Laboratory Animal Technology Co., Ltd. (Beijing, China). All animal procedures were reviewed and approved by the Animal Ethics Committee of Wenzhou Institute, University of Chinese Academy of Sciences (WIUCAS25060404). BALB/c nude and BALB/c mice (aged 7 weeks) were obtained from Beijing Vital River Laboratory Animal Technology Co., Ltd. (Beijing, China). For subcutaneous tumour xenograft models, U14 cells transfected with either VE-ATP7A or OE-ATP7A were suspended in PBS and injected subcutaneously (5 × 10⁶ cells in 100 μL) into the anterior right hind limb of BALB/c or BALB/c nude mice at a density of 5 × 10⁶ cells. Cisplatin (2 mg/kg) was injected intraperitoneally every two days when the tumour volume reached ~50 mm³. Tumour growth was monitored every two days, and tumours were collected for further analyses when the volume reached approximately 900 mm³ or when the maximum diameter was approximately 1.5 cm.

### Immunohistochemistry

Cervical cancer tissue samples and normal cervix tissue samples were obtained from the Second Affiliated Hospital of Wenzhou Medical University. Mouse tumour tissues were acquired from the animal experiments conducted in this study. Paraformaldehyde (4%) was used to fix the human cervical cancer and mouse tumour tissues. The paraffin waxes were embedded and cut into 5 μm-thick sections. Microwave-assisted antigen retrieval was performed using sodium citrate buffer (pH 6.0) after paraffin removal and rehydration. After a 1-hour blocking step with 5% BSA (Solarbio, China) at room temperature, the sections were subjected to overnight incubation at 4 °C with primary antibodies (including DLAT (#12362) from Cell Signaling Technology (USA), ATP7A (DF8506), LIAS (PH2927) from Affinity Biosciences (China), and FDX1 (ab108257) from Abcam (UK). CD8α antibody (sc-7970) was obtained from Santa Cruz Biotechnology (USA). Afterwards, the sections were incubated with HRP-conjugated goat anti-rabbit or anti-mouse IgG secondary antibodies (ZSGB-BIO, PV-9001/PV-9002, China) for 40 min at 37 °C. Following visualization of immunoreactivity via a DAB detection kit (3,3′-diaminobenzidine; ZSGB-BIO, ZLI-9018, China), the sections were counterstained with haematoxylin for 20 s. The immunoreactivity score was computed as the product of the staining intensity score (0 (negative), 1 (weak), 2 (moderate), 3 (strong)) and the percentage of reactive cells (0 = 0%, 1 ≤ 25%, 2 = 25–50%, 3 = 51–75%, 4 ≥ 75%). The final IRS values were used for statistical analysis.

### Immunofluorescence imaging

For mouse tumour tissue sections, the same procedure was followed for immunohistochemistry, followed by incubation with primary antibodies (P-gp (#13978) from Cell Signaling Technology (USA) and CD8α (sc-7970) from Santa Cruz Biotechnology (USA)) at 4 °C for 14 h and fluorescent secondary antibodies (Abcam, ab186697/ab150106, UK) at 37 °C for 2 h at room temperature. DAPI mounting medium (Abcam, UK) was used to counterstain the nuclei. The fluorescence was subsequently captured using a confocal microscope.

### Tumour interstitial fluid collection

Tumour interstitial fluid (TIF) was collected from freshly excised mouse tissues within 30 min after resection. Briefly, tumour tissues were rinsed three times with ice-cold PBS, minced into small fragments, and centrifuged at 2000 rpm for 20 min at 4 °C. After centrifugation (or relevant pretreatment), the supernatant was harvested and filtered through a 20 μm nylon mesh to obtain clarified TIFs.

### Tumour tissue dissociation and treatment

The tumour tissues were processed into single-cell suspensions with a tumour tissue dissociation kit (Yuanxin Biotechnology, China) following the manufacturer’s instructions. Approximately 0.5–1 g of freshly collected tumour tissue was minced to remove necrotic and fatty areas and then digested with the enzyme mixture at 37 °C and 150 rpm for 30 min. The digested suspension was filtered through a 40 μm cell strainer, and red blood cells were lysed using RBC lysis buffer. After centrifugation, the resulting single-cell pellet was resuspended in complete medium and incubated for 6 h at 37 °C in a 5% CO₂ incubator with a cell activation cocktail (BioLegend, 423304, USA), after which the treated cells were subsequently collected for flow cytometric analysis.

### Inductively coupled plasma–mass spectrometry

Copper ion concentrations were quantified in cells, cell culture supernatants, and TIFs using ICP–MS. Briefly, samples were collected and digested with aqua regia (concentrated hydrochloric acid: concentrated nitric acid, 3:1) at 200 °C. Copper ion contents were determined using an ICP–MS system (Agilent 7900, USA) calibrated with certified standard solutions.

### Flow cytometry

The cells were incubated with surface marker antibodies (including anti-CD8α-PE (#100709), anti-CD25-APC (clone M-A251, #302609/356109), anti-CD44-FITC (clone IM7, #338803), anti-CD69-BV750 (clone FN50, #19061), and the Zombie NIR™ Fixable Viability Kit (#423106) from BioLegend (USA) at 37 °C for 1 h, followed by fixation/permeabilization buffer (BD Biosciences, 554722, USA). Subsequently, the intracellular cytokines were stained with specific antibodies (including IFN-γ-FITC (#502507) and GzmB-PerCP-Cy5.5 (#372212) from BioLegend (USA)) at 37 °C for 1 h.

Apoptosis of tumour cells was evaluated. Nonfluorescent tumour cells were stained using an Annexin V-FITC/PE apoptosis detection kit (BD Biosciences, 556547, USA), while fluorescent tumour cells were analysed with an Annexin V-7-AAD/PI apoptosis detection kit (BD Biosciences, 559763, UK). After staining, the cells were incubated for 15 min at room temperature in the dark before data acquisition.

Single-cell suspensions were collected from mouse tumour tissue as described earlier. After resuspension in staining buffer, anti-mouse CD16/32 antibody (BioLegend, 101301, USA) was used to block Fc receptors through incubation for 30 min at 4 °C. Afterwards, the cells were stained with the appropriate antibody panels (including anti-CD3-APC (#100236), anti-CD45-BV510 (#103138), anti-CD8α-PE (#100708), anti-CD25-APC (#102012), anti-CD44-FITC (#103006), and anti-CD69-BV750 (#104548) from BioLegend (USA)) at 4 °C for 1 h after the application of fixation/permeabilization buffer (BioLegend, 554722, USA). Intracellular cytokine staining (IFN-γ-FITC (#505806) and GzmB-PerCP-Cy5.5 (#372212) from BioLegend (USA)) was performed at 4 °C for 1 h. All the samples were assessed on a CytoFLEX LX flow cytometer (Beckman Coulter, USA), and FlowJo software was used to process the data (v10.8.1; Tree Star, USA).

### Molecular docking and molecular dynamics simulations

Molecular docking was performed using AutoDock Vina to examine the interactions between Cu²⁺ and LCK and between SOX21 and ATP7A. The crystal structures of the proteins were obtained from the Protein Data Bank. All water molecules and ligands were removed before docking. Docking grids were centred on the LCK activation loop (Tyr394) and the ATP7A promoter-binding region predicted by JASPAR. The lowest-energy conformations were visualized in PyMOL and chosen for molecular dynamics simulations. GROMACS (version 2023.2) with the CHARMM36 force field was used to perform molecular dynamics simulations. The Cu²⁺-LCK complex was dissolved in a TIP3P water box with 150 mM NaCl. Utilizing the Parrinello-Rahman barostat and V-rescale thermostat, a 50 ns production run was performed at 310 K and 1 bar with a 2 fs timestep after energy minimization and NVT/NPT equilibration (100 ps each). Trajectory analyses were performed after the periodic boundary artefacts were removed. The RMSD, RMSF, and Cu²⁺-Tyr394 distance were computed to assess conformational stability and dynamics using GROMACS.

### Statistics

R software (version 4.3.2), GraphPad Prism (version 9.0), and SPSS (version 26.0) were used to perform all the statistical analyses. Continuous data are expressed as the mean ± SD and were compared using the *t*-test or one-way ANOVA with Tukey’s post hoc test; nonparametric tests were applied when appropriate. Correlations were evaluated by Spearman’s or Pearson’s analysis. Survival analyses were performed using the Kaplan–Meier method, and the data were compared by the log-rank test. A two-sided *P* < 0.05 was considered to indicate statistical significance. Human sample sizes for in vitro (*n* = 10/group) were selected according to prior studies in the field. These values ensured adequate power ( ≥ 80%) to detect anticipated intergroup differences at α = 0.05, while balancing statistical validity with ethical constraints to reduce animal numbers. No sample size estimate, randomization or blinding were used in animal studies.

## Supplementary information

Supplemental Figure

Supplemental Table

Western blot

## Data Availability

The datasets generated during and/or analysed during the current study are available from the corresponding author upon reasonable request.
