# Supplementary material for: ATP7A-mediated copper ion efflux reprogrammes tumour immunity and promotes cisplatin resistance in cervical cancer
Source: Cell Death Dis. 2026 Jul 10;17(1):859. doi: 10.1038/s41419-026-09082-5 (PMC13639005; doi:10.1038/s41419-026-09082-5)

Figure 1J

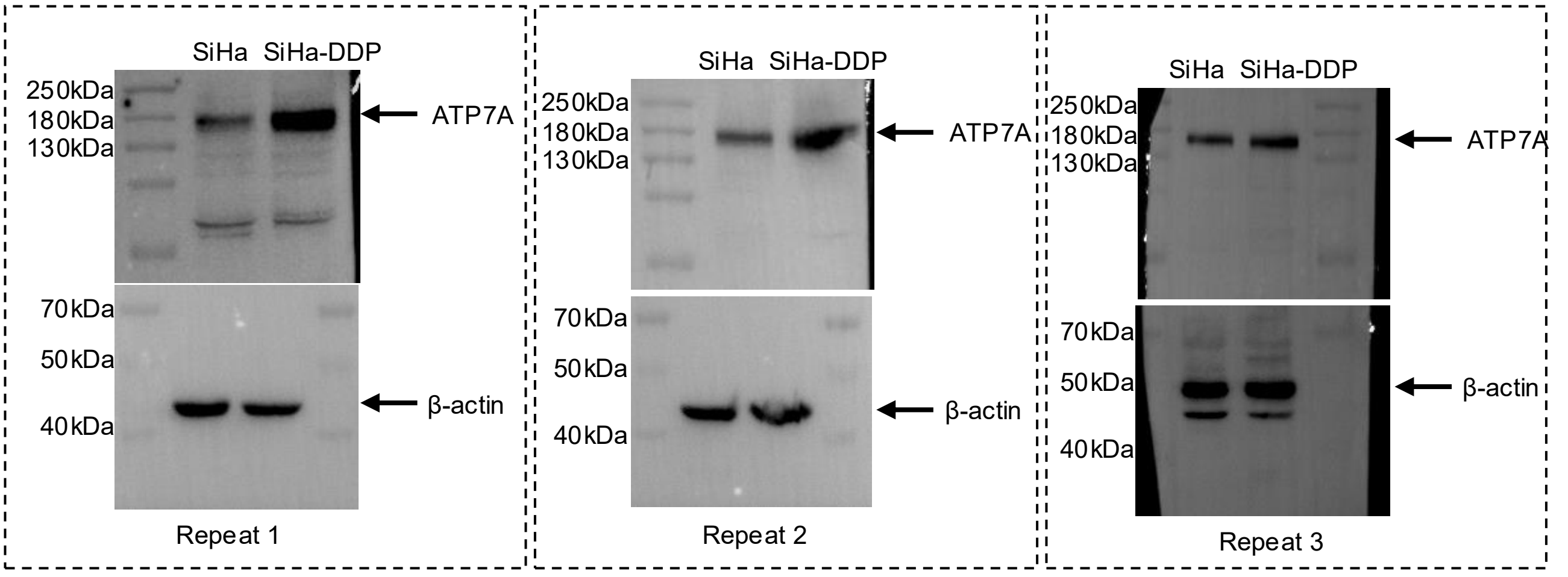

Figure 1N

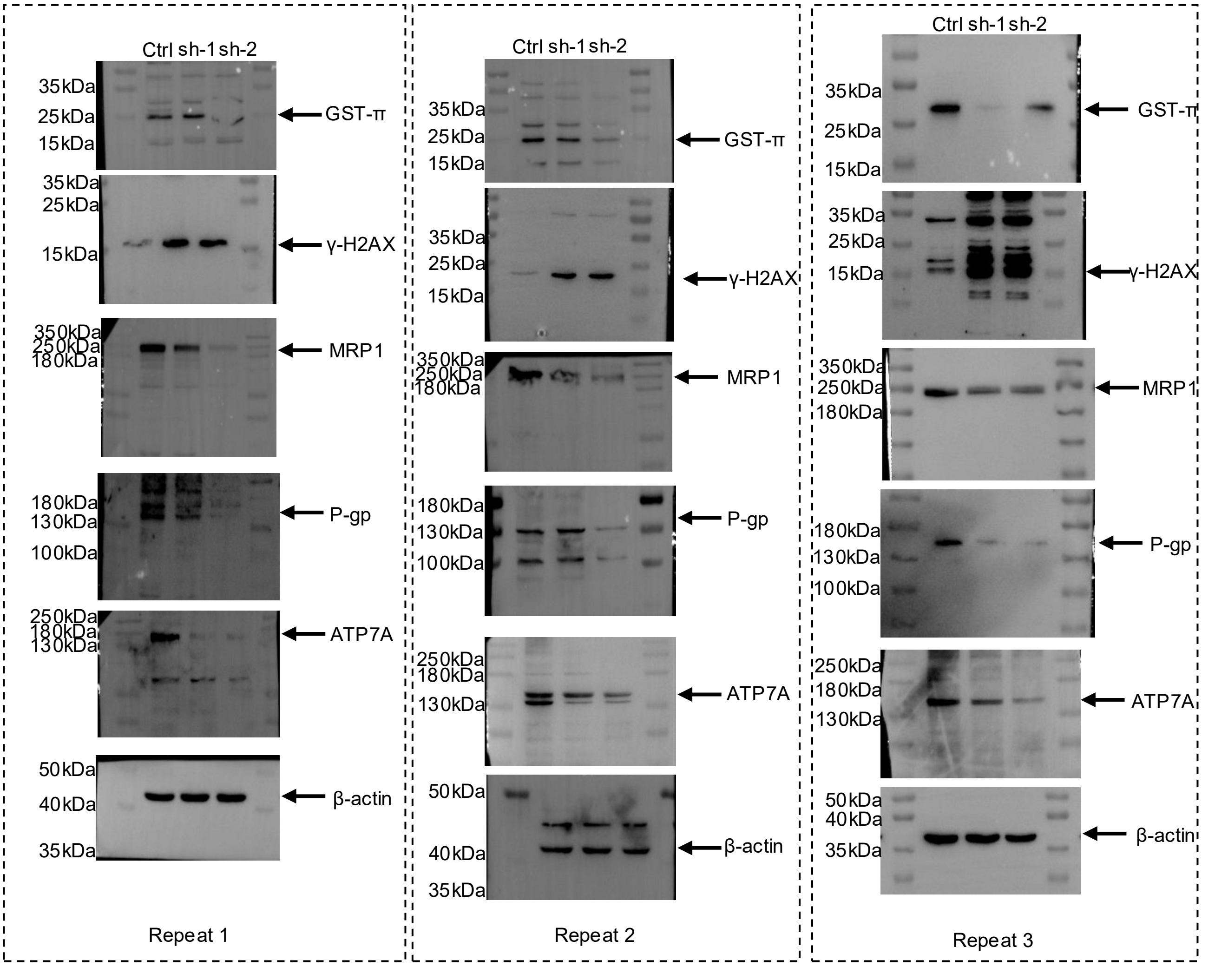

Figure 1O

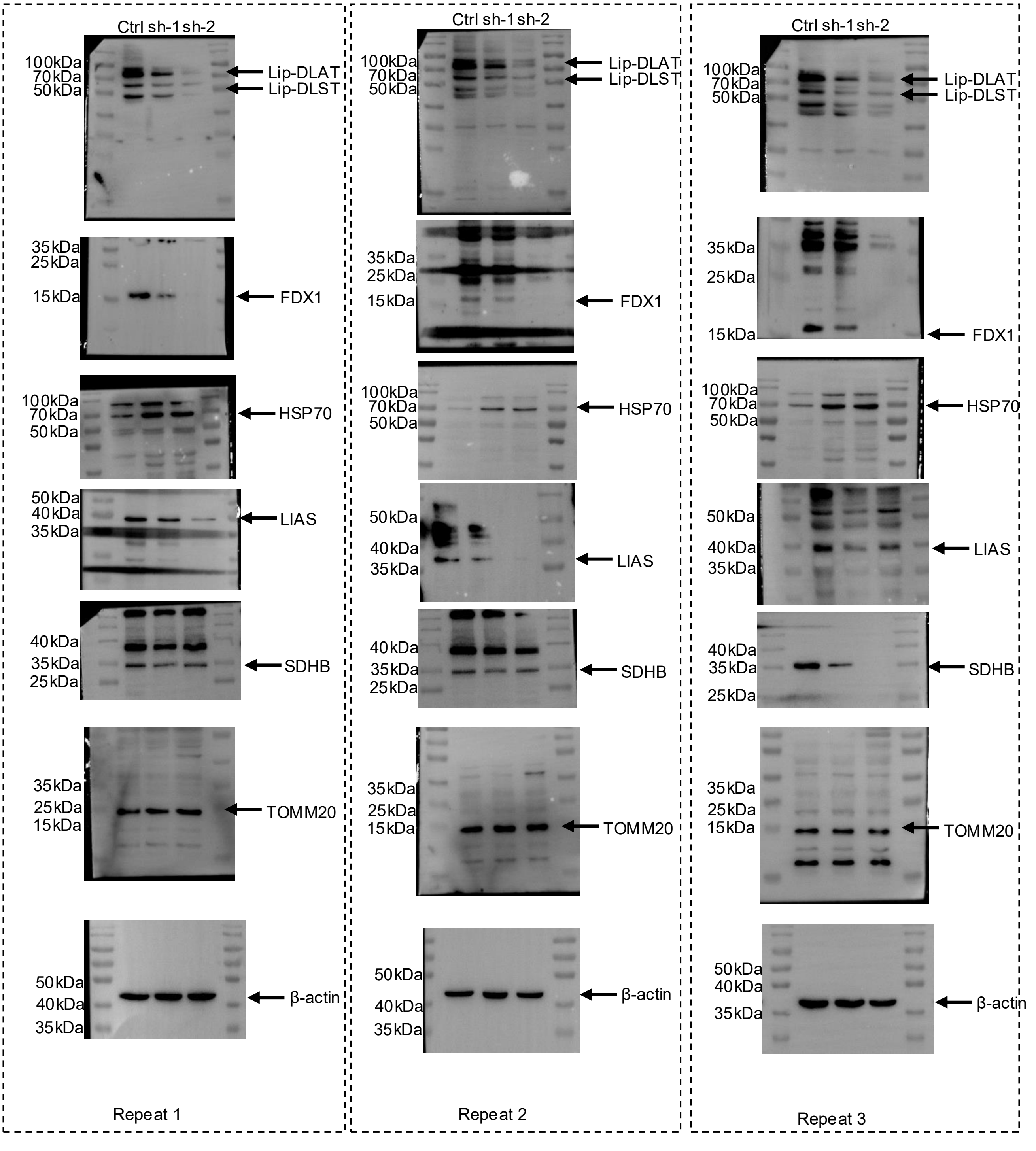

Ctrl sh-1 sh-2
100kDa
70kDa
50kDa
Lip-DLAT
Lip-DLST
35kDa
25kDa
15kDa
FDX1
HSP70
40kDa
LIAS
SDHB
TOMM20
β-actin
Repeat 1
Repeat 2
Repeat 3

Figure 5J

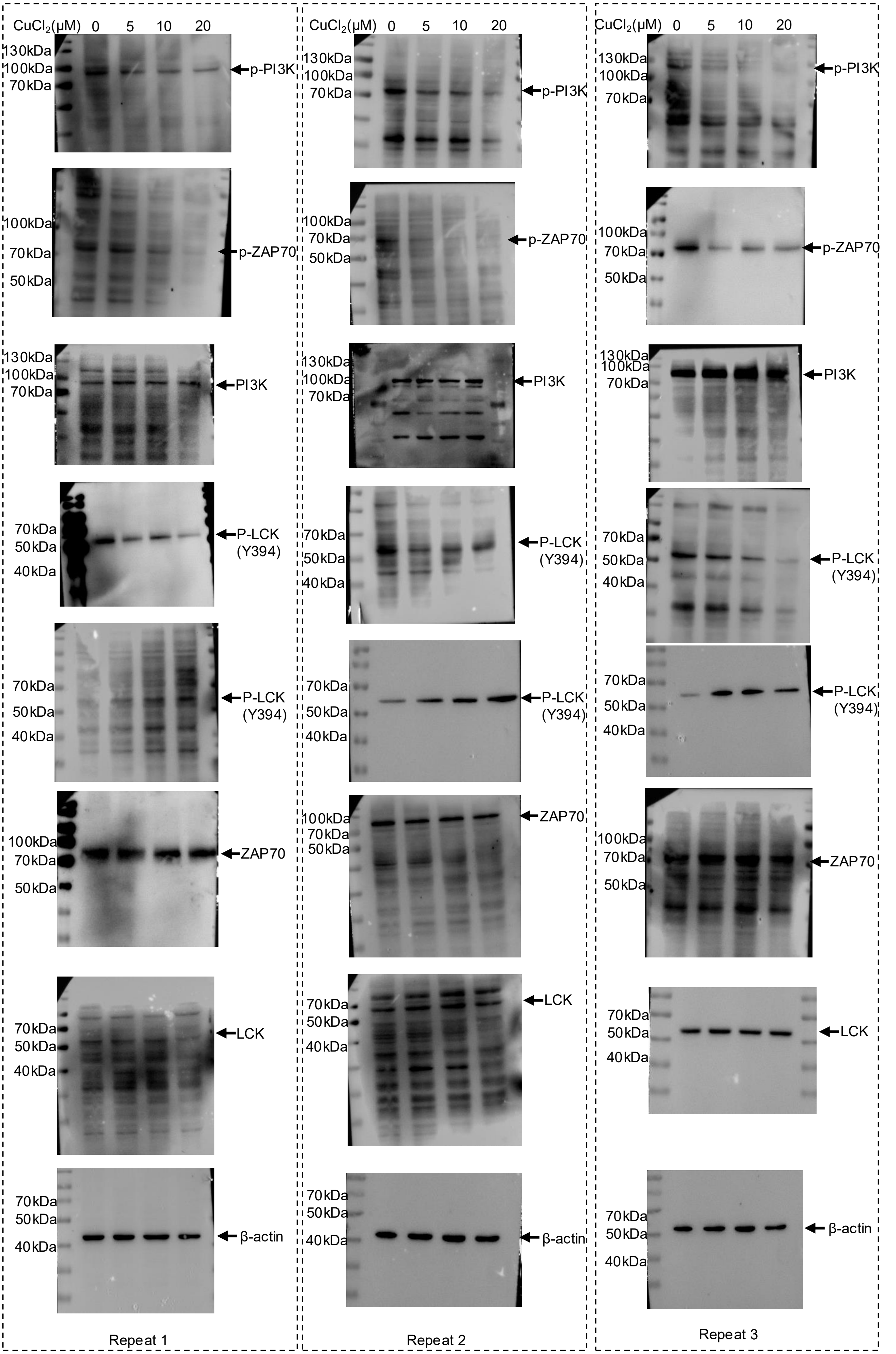
CuCl$_2$(μM) 0 5 10 20
130kDa
100kDa
70kDa
50kDa
40kDa
p-PI3K
p-ZAP70
PI3K
P-LCK (Y394)
ZAP70
LCK
β-actin
Repeat 1
Repeat 2
Repeat 3

Figure 5K

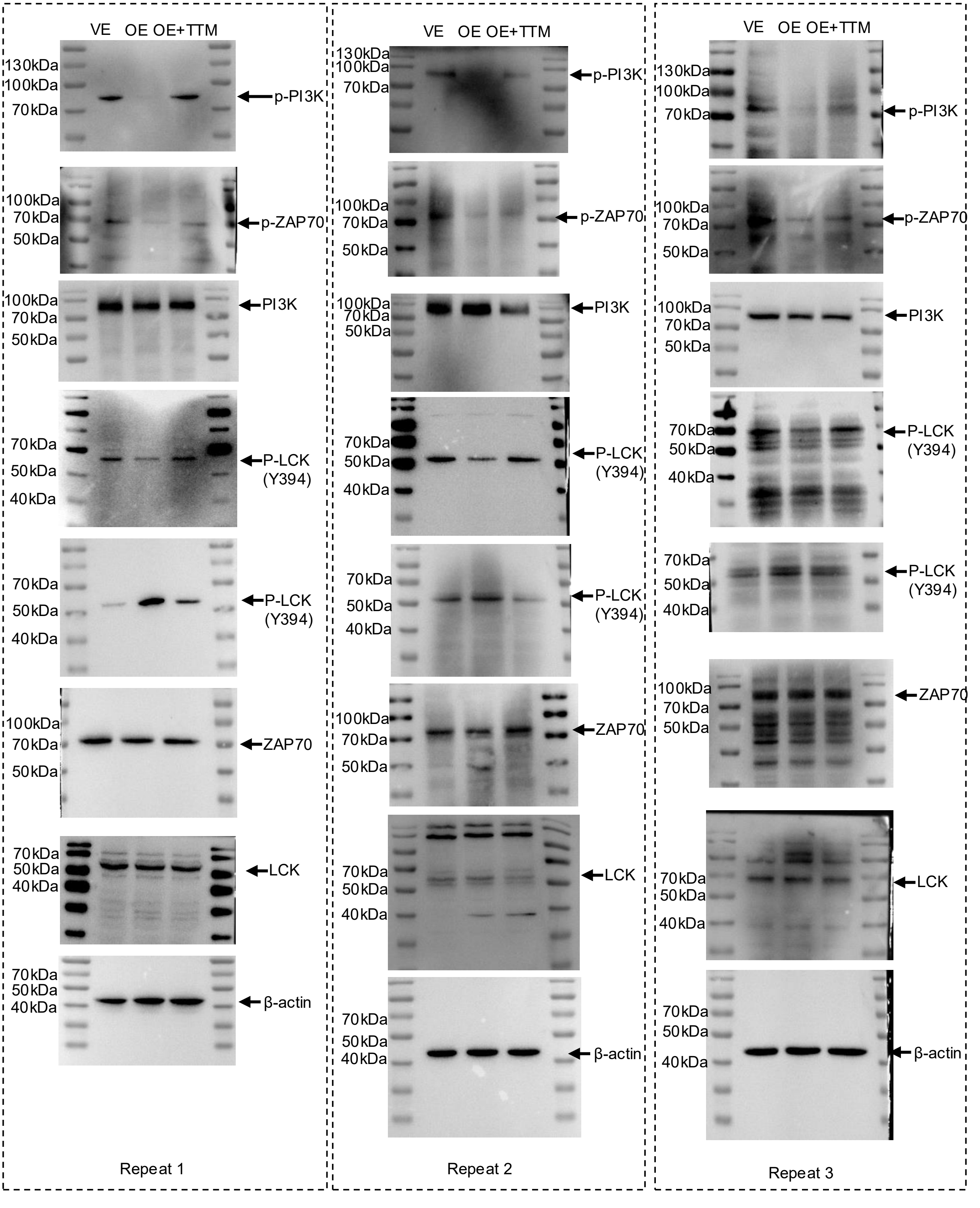
VE
OE
OE+TTM
130kDa
100kDa
70kDa
p-PI3K
p-ZAP70
50kDa
PI3K
P-LCK
(Y394)
40kDa
ZAP70
LCK
β-actin
Repeat 1
Repeat 2
Repeat 3

Figure 5O

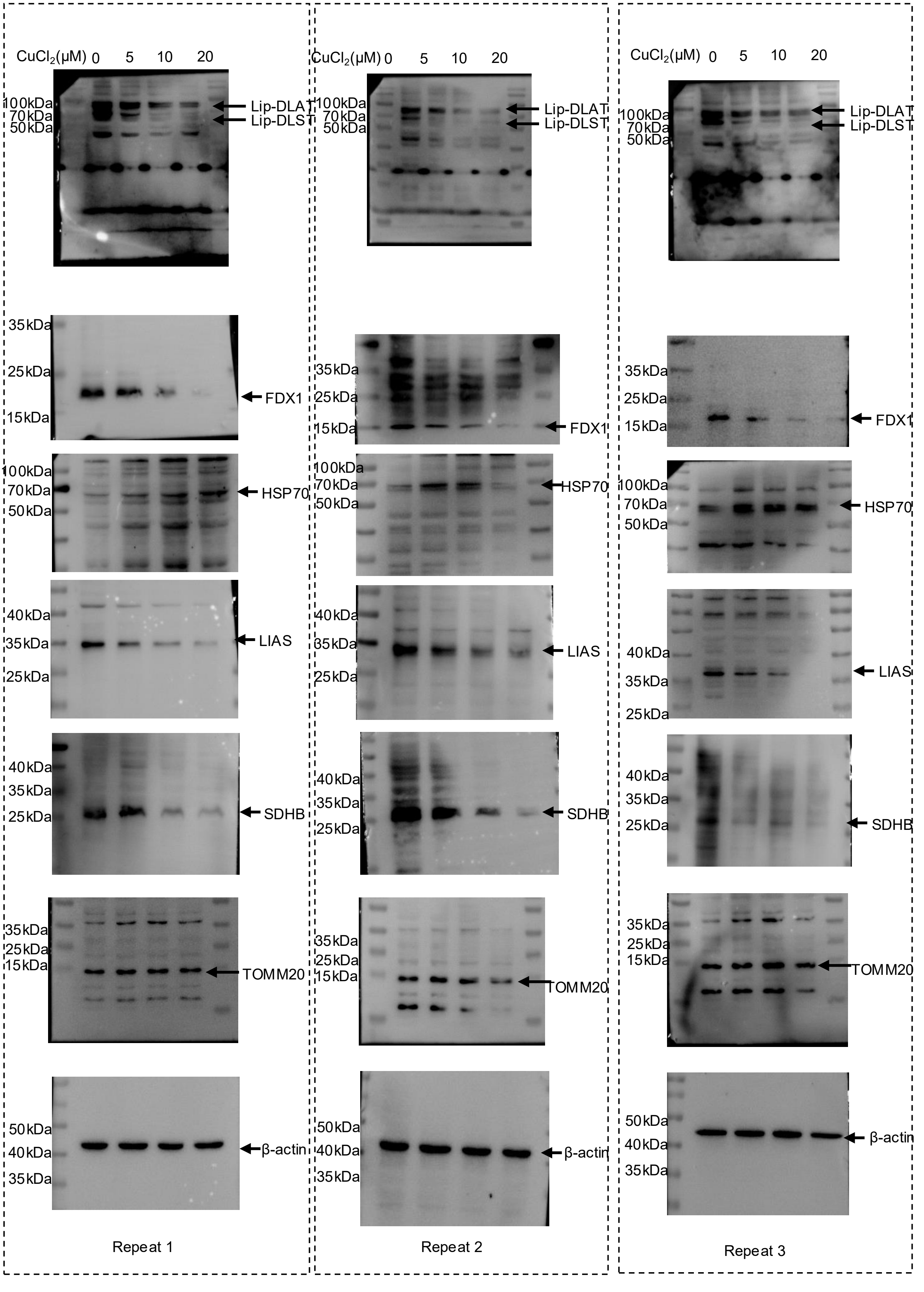

CuCl2(μM) 0 5 10 20
100kDa
70kDa
50kDa
Lip-DLAT
Lip-DLST
35kDa
25kDa
15kDa
FDX1
HSP70
40kDa
LIAS
SDHB
TOMM20
β-actin
Repeat 1
Repeat 2
Repeat 3

Figure 5Q

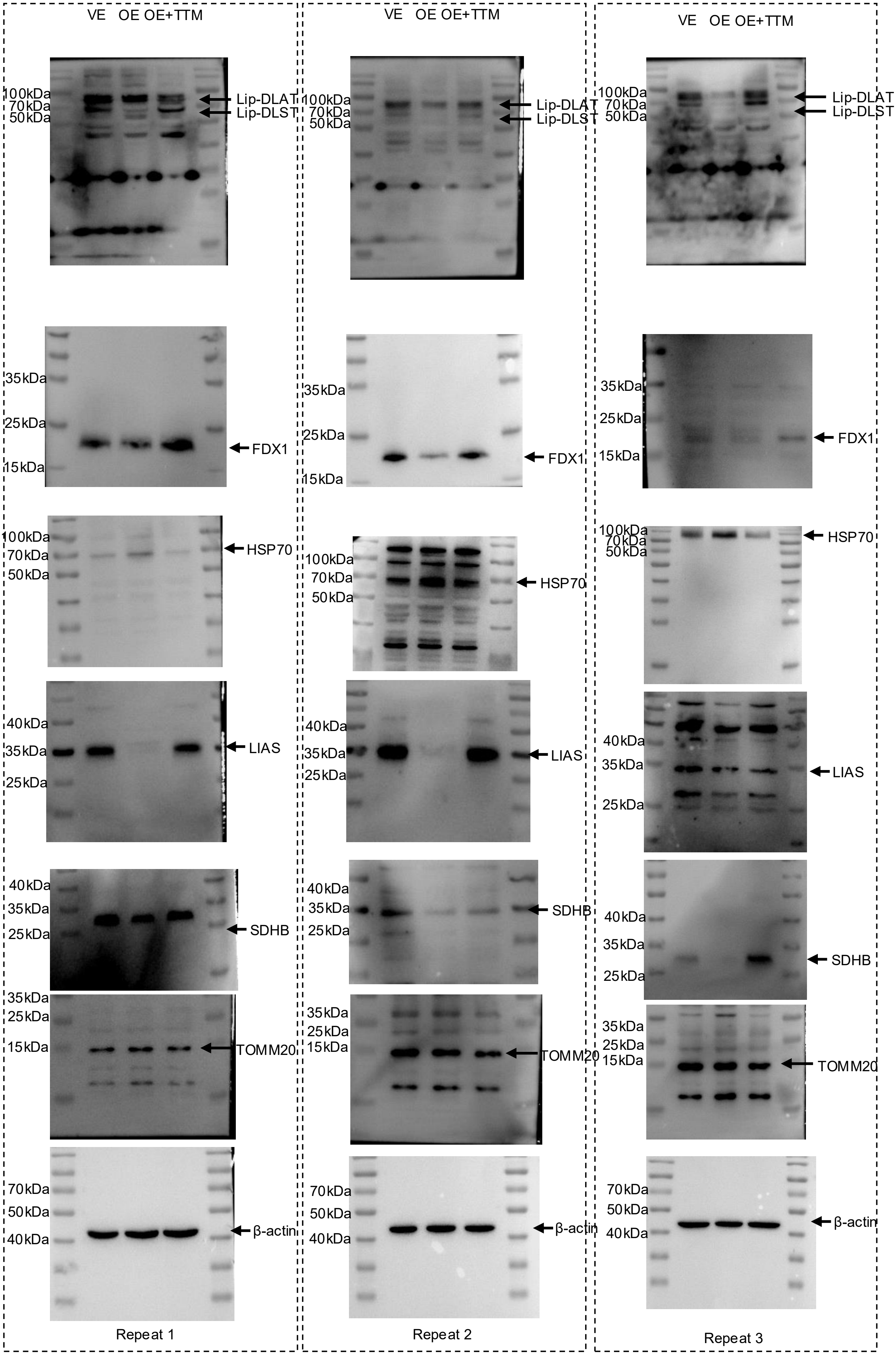

VE OE OE+TTM
100kDa
70kDa
50kDa
Lip-DLAT
Lip-DLST
35kDa
25kDa
15kDa
FDX1
100kDa
70kDa
50kDa
HSP70
40kDa
35kDa
25kDa
LIAS
40kDa
35kDa
25kDa
SDHB
35kDa
25kDa
15kDa
TOMM20
70kDa
50kDa
40kDa
β-actin
Repeat 1
VE OE OE+TTM
100kDa
70kDa
50kDa
Lip-DLAT
Lip-DLST
35kDa
25kDa
15kDa
FDX1
100kDa
70kDa
50kDa
HSP70
40kDa
35kDa
25kDa
LIAS
40kDa
35kDa
25kDa
SDHB
35kDa
25kDa
15kDa
TOMM20
70kDa
50kDa
40kDa
β-actin
Repeat 2
VE OE OE+TTM
100kDa
70kDa
50kDa
Lip-DLAT
Lip-DLST
35kDa
25kDa
15kDa
FDX1
100kDa
70kDa
50kDa
HSP70
40kDa
35kDa
25kDa
LIAS
40kDa
35kDa
25kDa
SDHB
35kDa
25kDa
15kDa
TOMM20
70kDa
50kDa
40kDa
β-actin
Repeat 3

Figure 6B

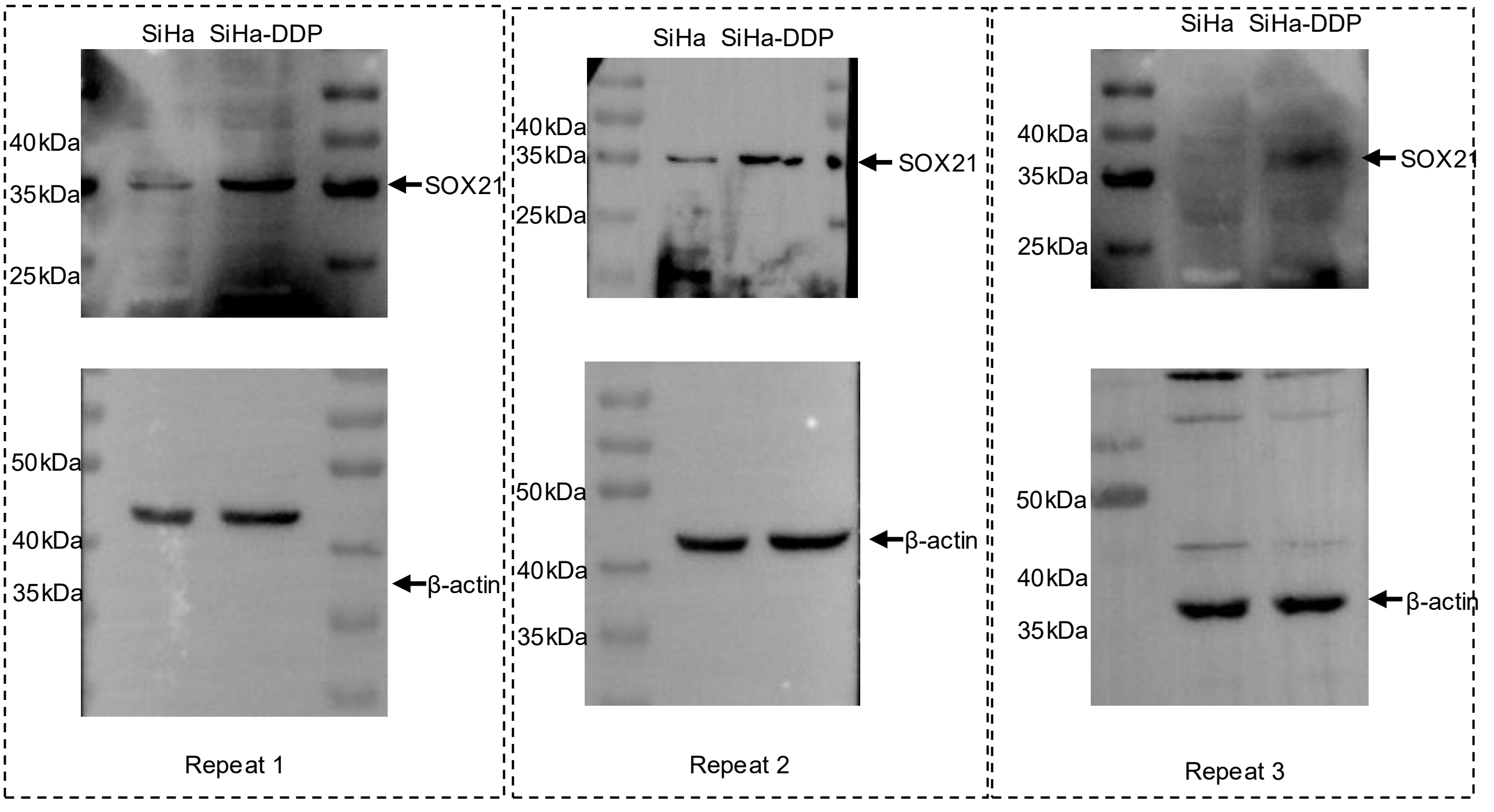

Figure 6F

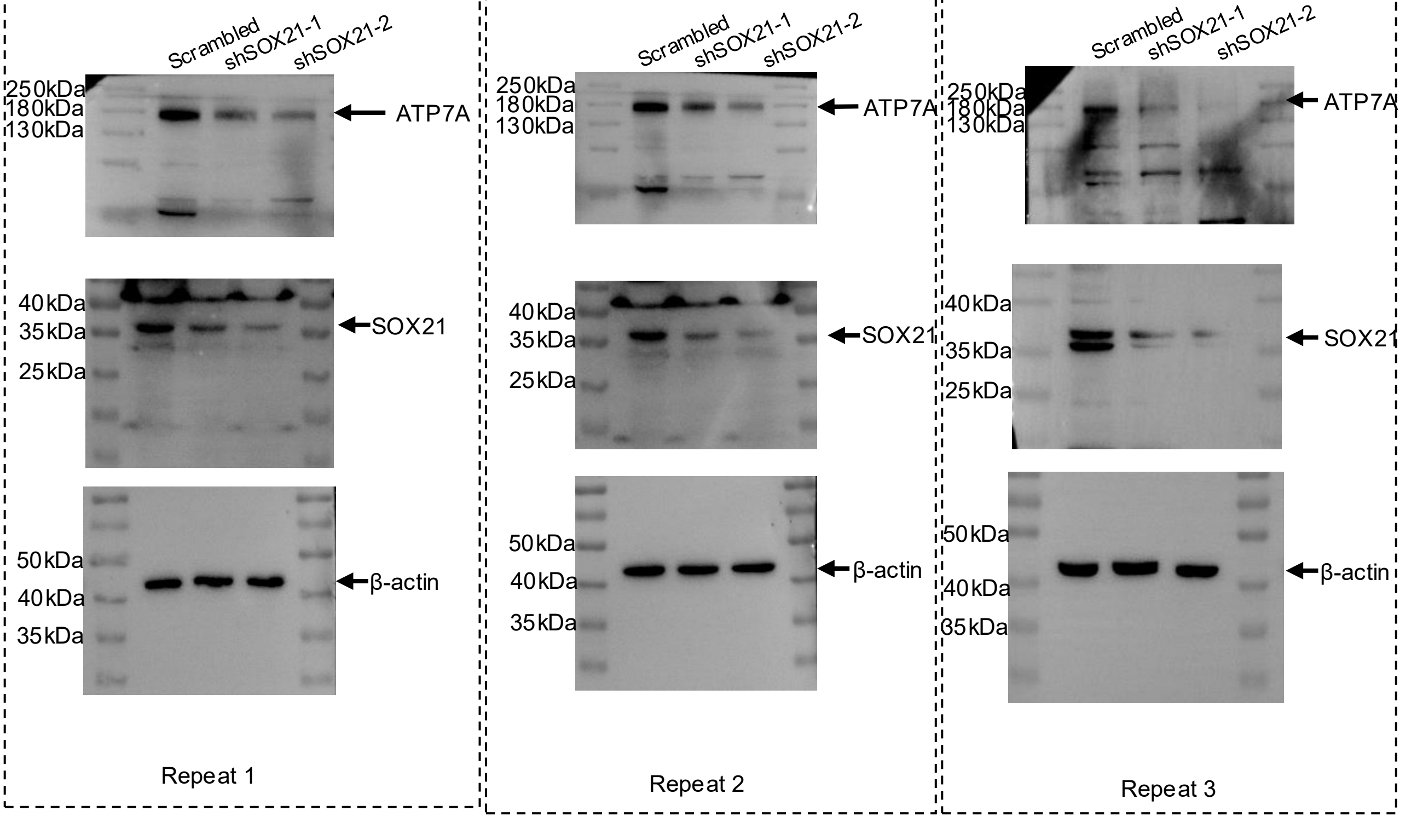

Figure 6L

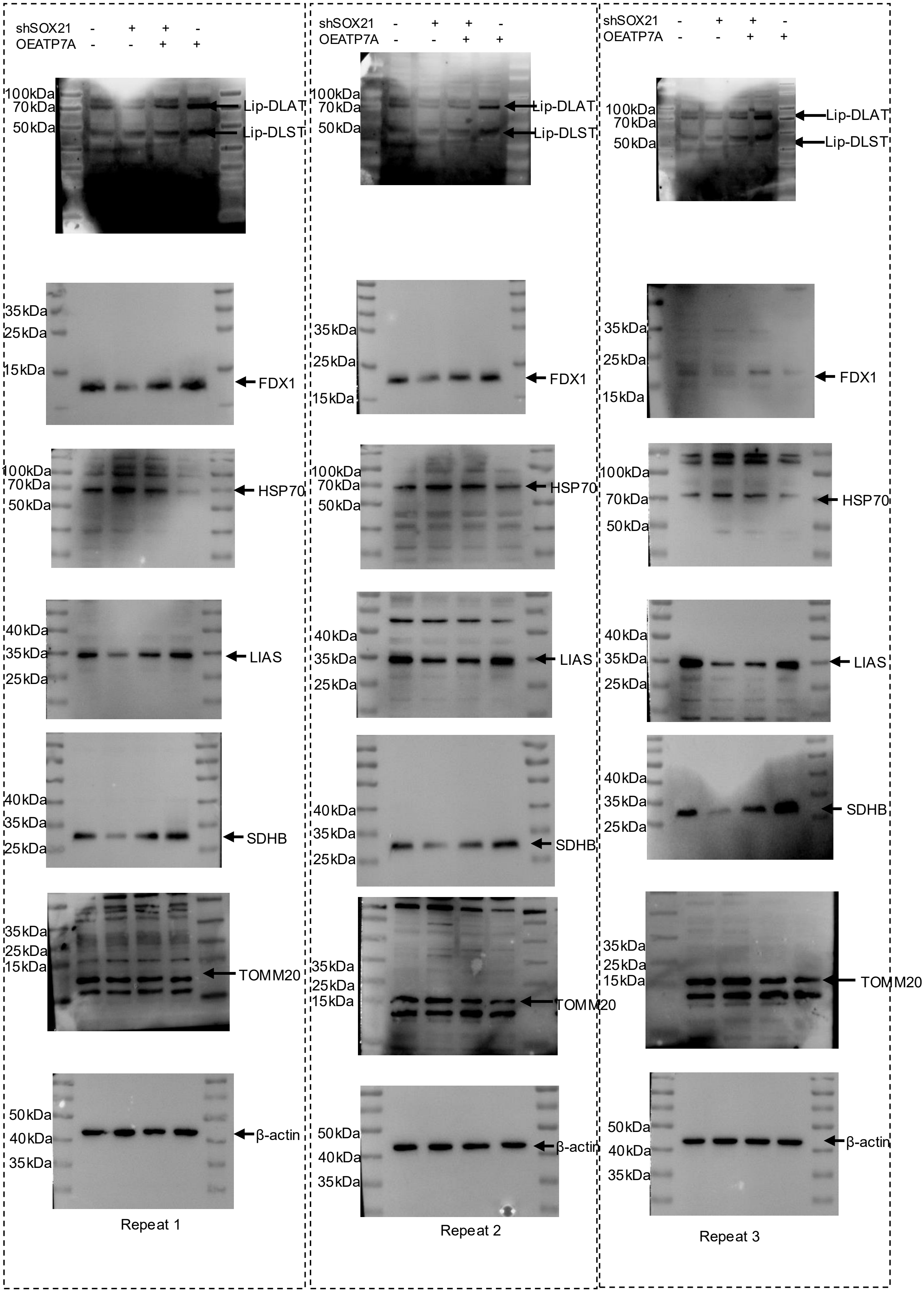

shSOX21 - + + -
OEATP7A - - + +
100kDa
70kDa
50kDa
Lip-DLAT
Lip-DLST
35kDa
25kDa
15kDa
FDX1
HSP70
40kDa
LIAS
SDHB
TOMM20
β-actin
Repeat 1
Repeat 2
Repeat 3

Figure S1C

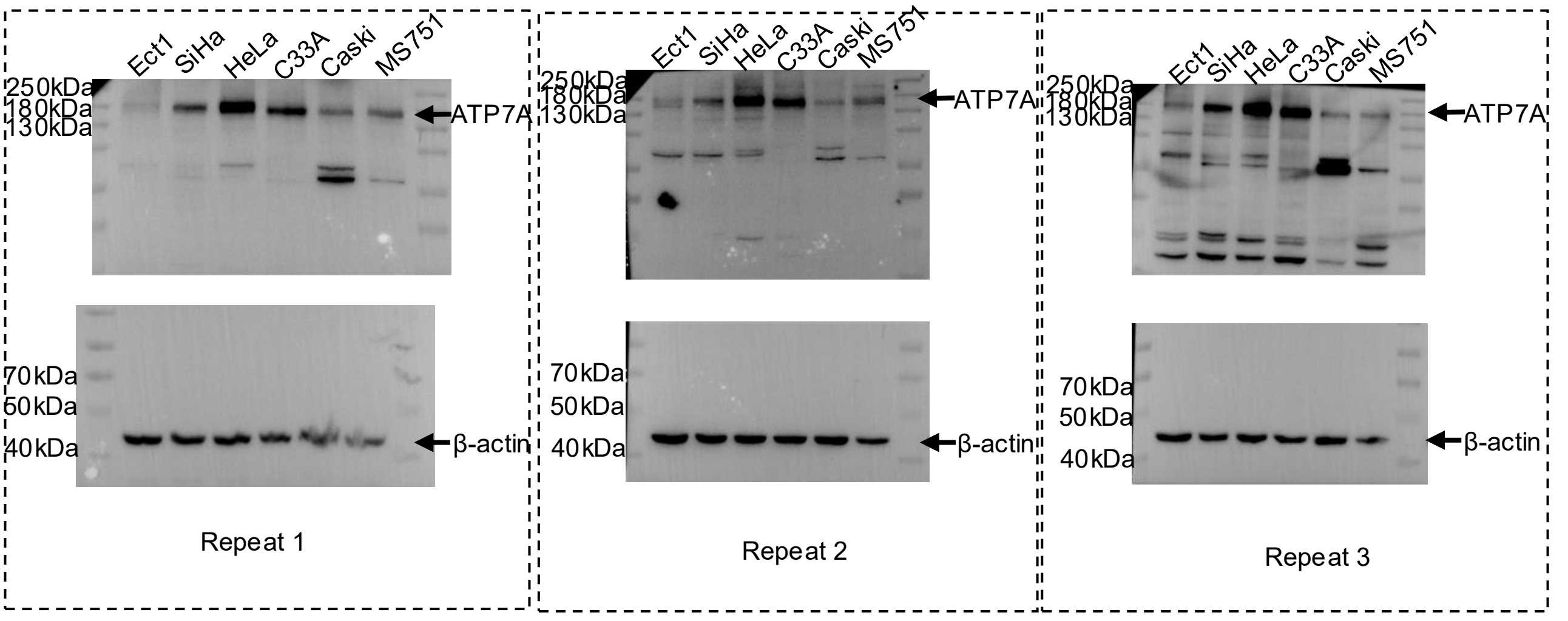
Ect1
SiHa
HeLa
C33A
Caski
MS751
250kDa
180kDa
130kDa
ATP7A
70kDa
60kDa
50kDa
40kDa
β-actin
Repeat 1
Repeat 2
Repeat 3

Figure S2A

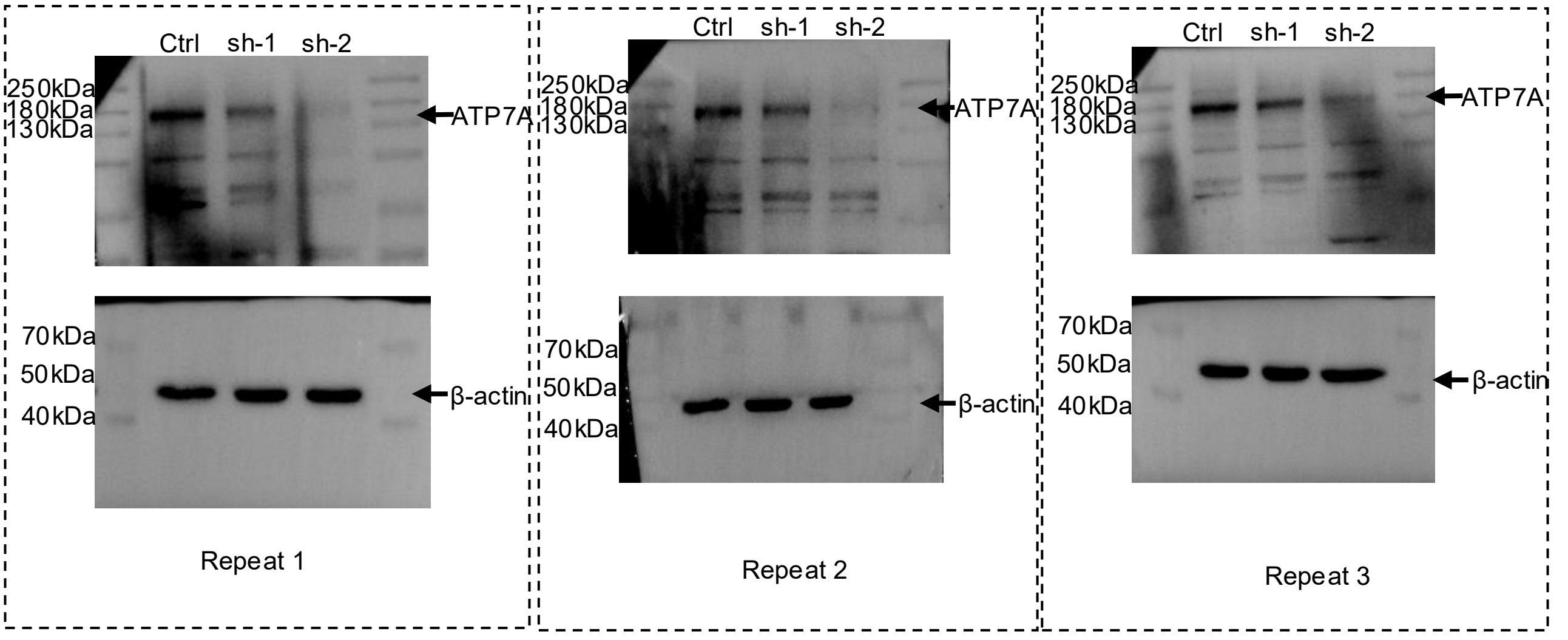

Figure S2C

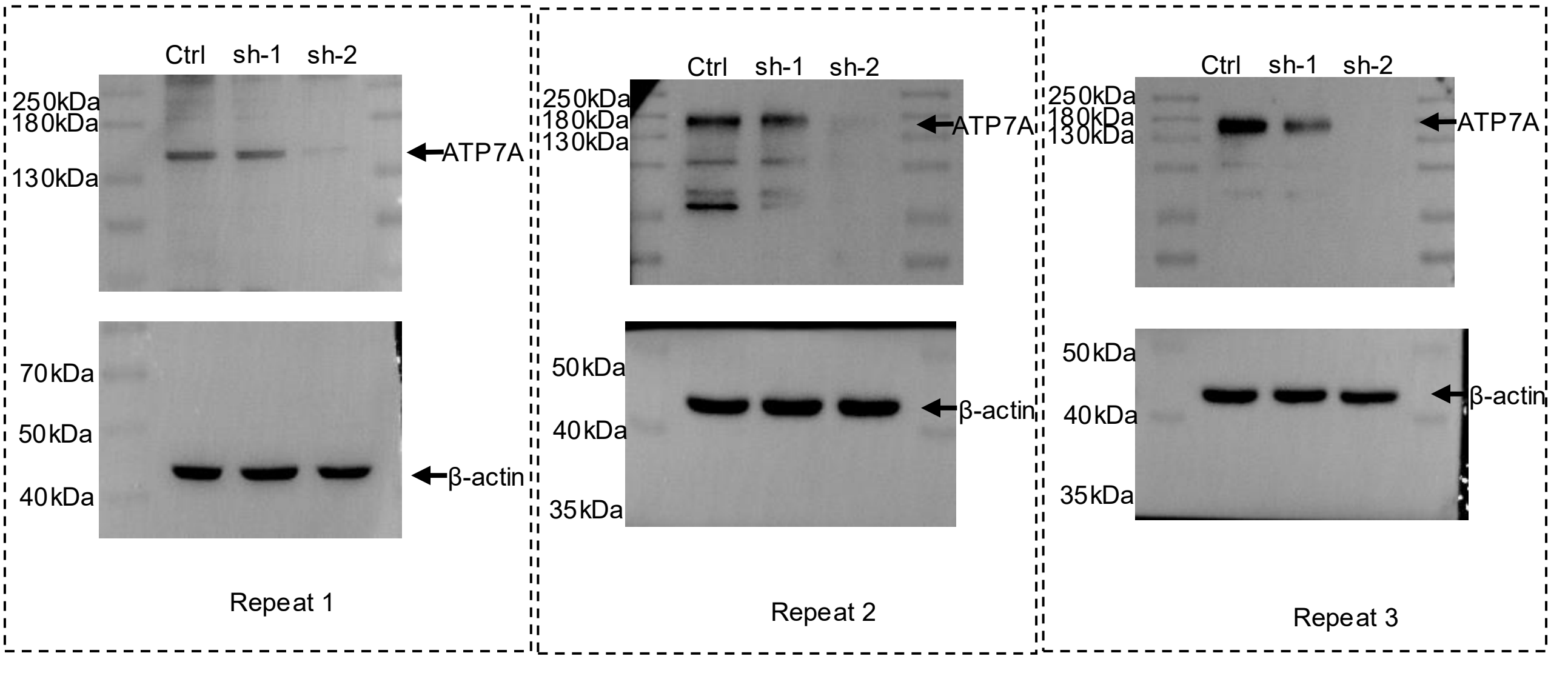

Figure S3C

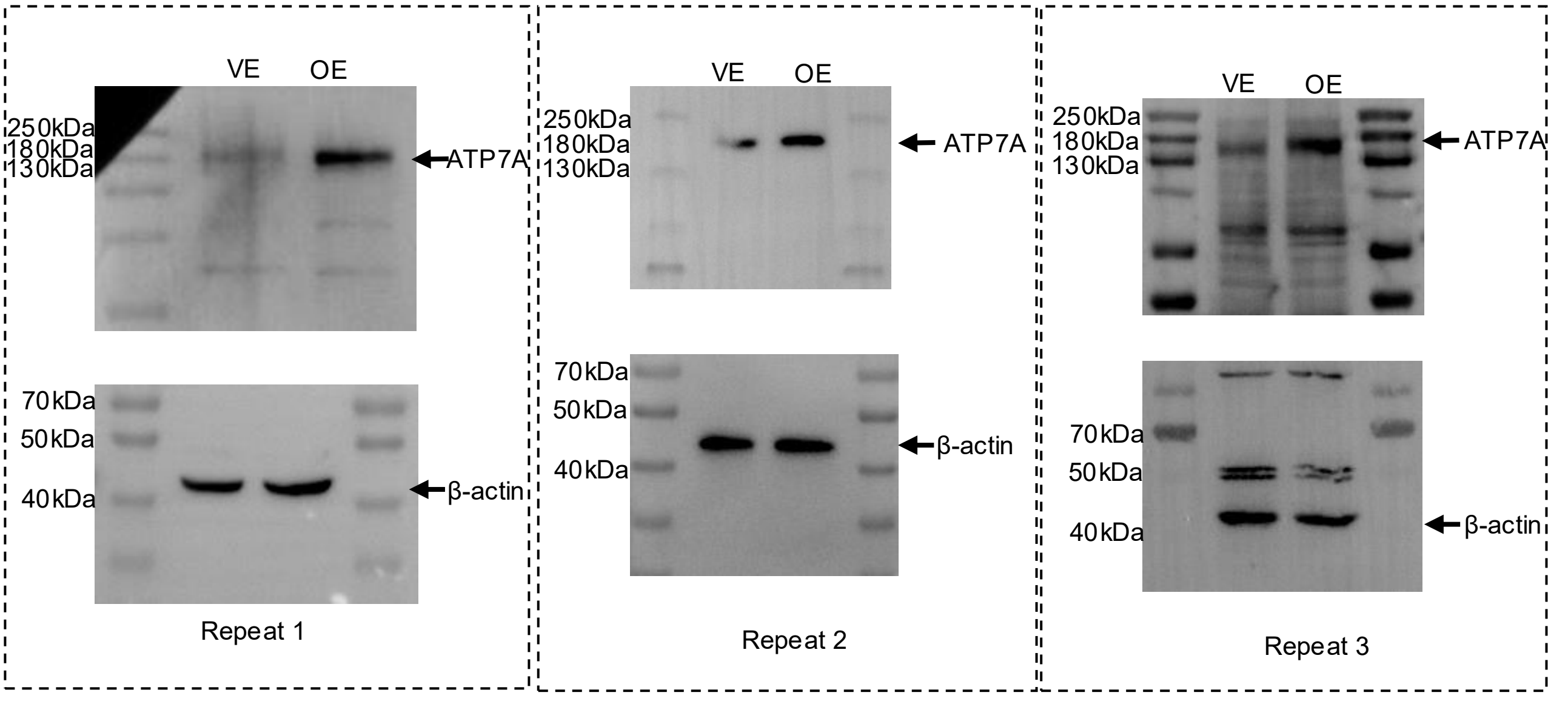

Figure S3D

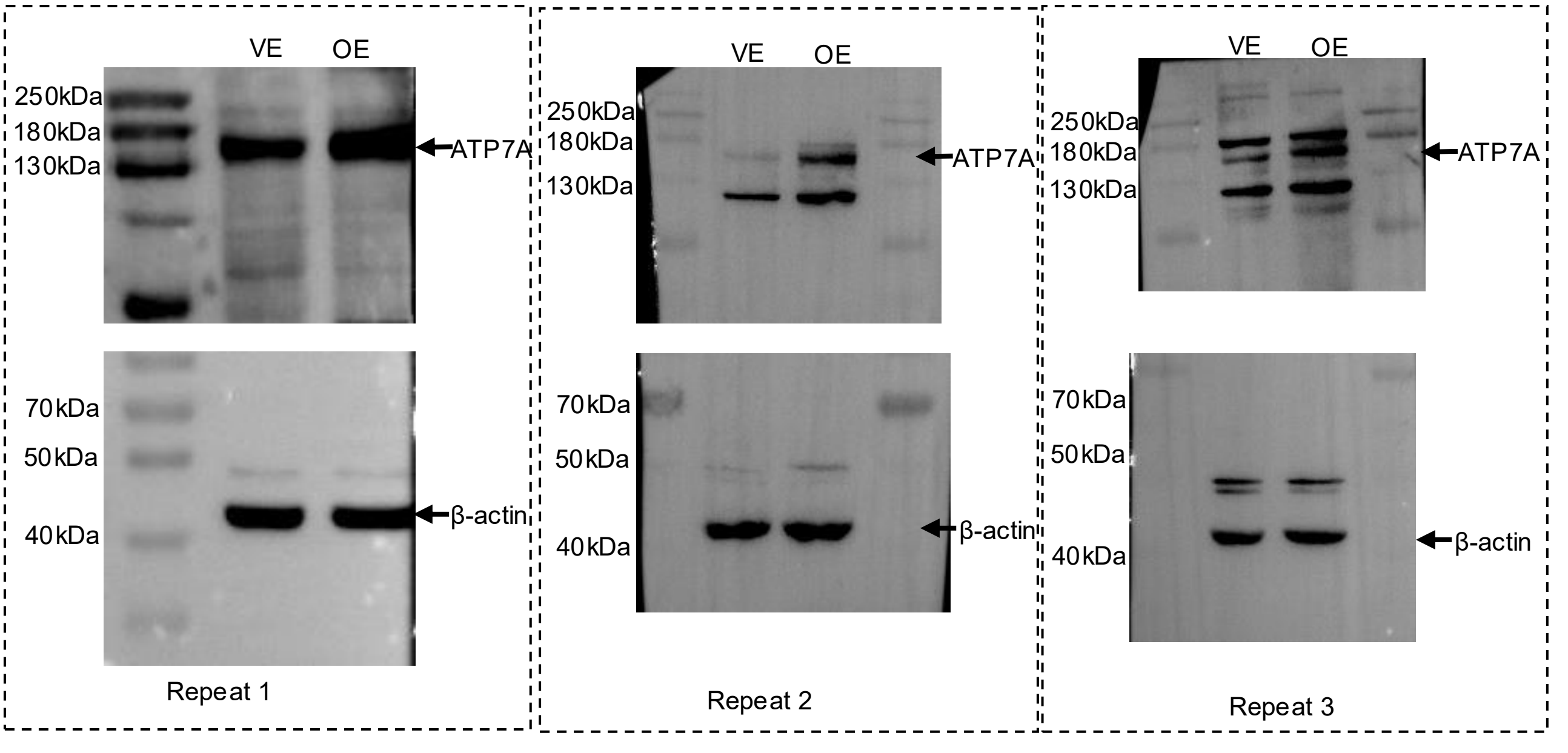

Figure S3H

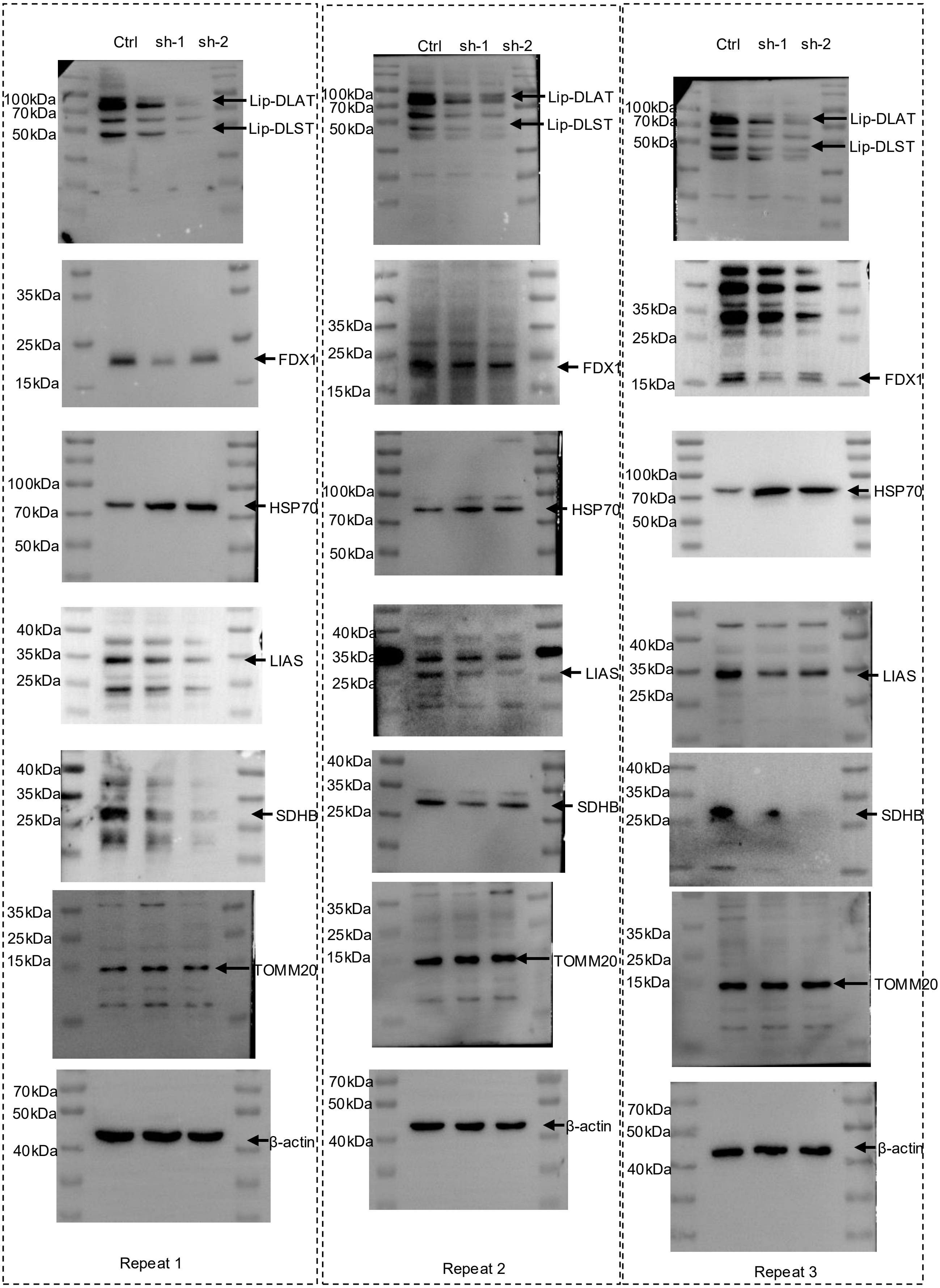

Ctrl
sh-1
sh-2
100kDa
70kDa
50kDa
Lip-DLAT
Lip-DLST
35kDa
25kDa
15kDa
FDX1
HSP70
40kDa
LIAS
SDHB
TOMM20
β-actin
Repeat 1
Repeat 2
Repeat 3

Figure S4C

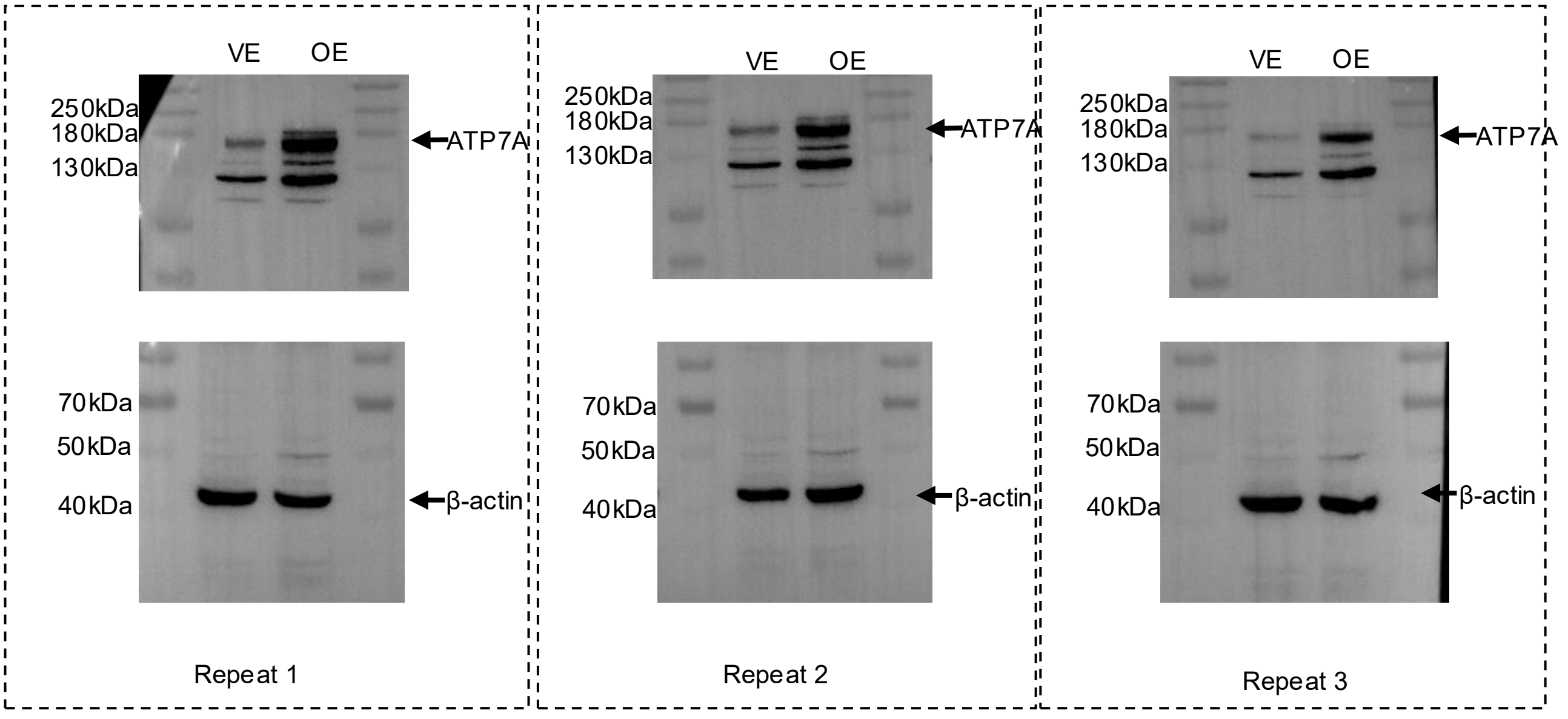

Figure S4D

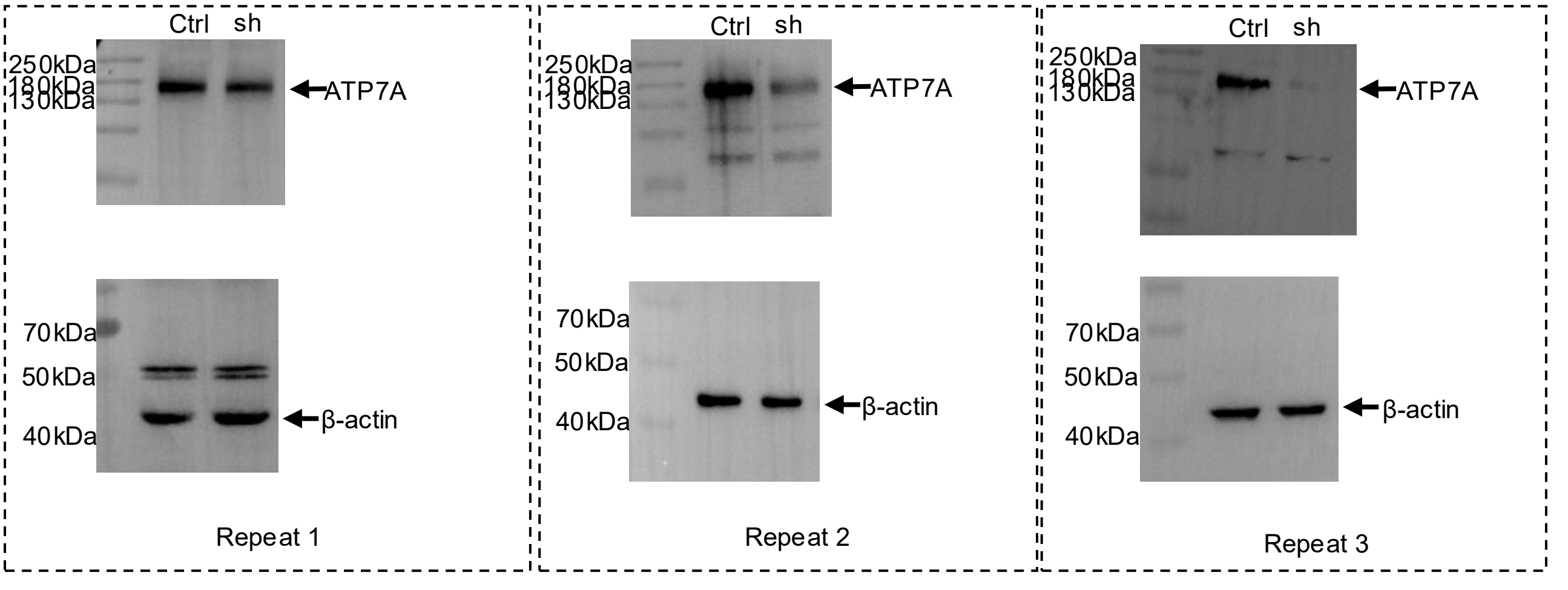

Supplement: Supplementary file 1 [file 41419_2026_9082_MOESM3_ESM.pdf]
