# Supplementary material for: ATP7A-mediated copper ion efflux reprogrammes tumour immunity and promotes cisplatin resistance in cervical cancer
Source: Cell Death Dis. 2026 Jul 10;17(1):859. doi: 10.1038/s41419-026-09082-5 (PMC13639005; doi:10.1038/s41419-026-09082-5)
Supplement: Supplementary file 2 [file 41419_2026_9082_MOESM1_ESM.docx]

Figure S1: (A) Determination of the optimal serum Cu cutoff in the NHANES 2011-2016 cancer cohort (n=459). (B) Kaplan-Meier survival analysis of patients with low and high ATP7A expression in cervical cancer cohort. (C) Western blot analysis of ATP7A protein levels in cervical cancer cell lines (SiHa, HeLa, C33A, Caski, MS751) and normal cervical epithelial cells (Ect1). (D) Stratification of TCGA-CESC cohort into cisplatin-sensitive and cisplatin-resistant groups. (E) ROC curves for Elastic Net and XGBoost models evaluating cisplatin resistance prediction. (F-G) CCK-8 assay showing cell viability of SiHa and SiHa-DDP cells treated with different concentrations of DDP.

Figure S2: The effects of ATP7A on development of SiHa and SiHa-DDP cells. (A) Western blot analysis confirming ATP7A knockdown efficiency in SiHa cells. (B) CCK-8 analysis of SiHa cell proliferation after ATP7A knockdown. (C) Western blot analysis confirming ATP7A knockdown efficiency in SiHa-DDP cells. (D) CCK-8 analysis of SiHa-DDP cell proliferation following ATP7A knockdown. (E-F) Colony formation assay and quantification of colony numbers in SiHa cells with indicated treatments. (G-H) Flow cytometry analysis of apoptosis in SiHa cells after ATP7A knockdown. (I-J) Colony formation assay and quantification of proliferation in ATP7A-knockdown SiHa-DDP cells. (K-L) Flow cytometry analysis of apoptosis in SiHa-DDP cells following ATP7A silencing.

Figure S3: Inhibition of ATP7A promoted cuproptosis and affected cisplatin resistance in cervical cancer cells. (A-B) Cell viability and drug sensitivity of SiHa cells treated with cisplatin (0, 1, 2, 4, 8, 16, 32, 64 µM) for 48 h determined by CCK-8 assay. (C-D) Western blot analysis confirming ATP7A overexpression in SiHa and SiHa-DDP cells. (E-F) CCK-8 assay assessing cisplatin response in SiHa (treated with cisplatin (0, 1, 2, 4, 8, 16, 32 µM) for 48 h) and SiHa-DDP (treated with cisplatin (0, 2, 4, 8, 16, 32, 64, 128 µM) for 48 h) cells overexpressing ATP7A. (G) Measurement of intracellular Cu²⁺ concentration in SiHa cells by colorimetric assay. (H) Western blot analysis of cuproptosis-related proteins (Lip-DLAT, Lip-DLST, FDX1, LIAS, SDHB, TOMM20, and HSP70) in SiHa cells with ATP7A knockdown. (I-J) The effect of TTM on cisplatin sensitivity in ATP7A-knockdown SiHa-DDP cells by CCK-8 and IC₅₀ analysis. Cells were pre-treated with 1µM TTM for 6h, and then treated with cisplatin (0, 2, 4, 8, 16, 32, 64, 128 µM) for 48 h.

Figure S4: (A-B) Correlation analysis between ATP7A expression and IFN-γ or GzmB expression. (C-D) Western blot analysis verifying ATP7A knockdown and overexpression in U14 cells. (E-F) Flow cytometry assay detecting intracellular IFN-γ expression in murine CD8⁺T cells co-cultured with U14 cells. (G-H) Flow cytometry assay measuring GzmB expression in murine CD8⁺T cells. (I-J) Analysis of murine CD8⁺T cell apoptosis after co-culture. (K-L) Proliferation assay of murine CD8⁺T cells co-cultured with U14 cells. (M) Cell viability assay determining copper ions tolerance in SiHa, SiHa-DDP, and CD8⁺T cells.

Figure S5: Effects of ATP7A modulation on CD8⁺ T-cell function in cisplatin-resistant cervical cancer cells. (A) Direct coculture system involving human CD8⁺ T cells and SiHa-DDP cells with ATP7A knockdown. (B-D) Flow cytometry analysis of IFN-γ and GzmB expression and apoptosis in CD8⁺ T cells cocultured with SiHa-DDP cells with ATP7A knockdown. (E-F) CFSE-based proliferation assay and cytotoxicity analysis of CD8⁺ T cells in the direct coculture system. (G) Diagram of the Transwell coculture setup used to assess the indirect effects of ATP7A on CD8⁺ T cells. (H-J) Flow cytometry analysis of cytokine production, proliferation, and apoptosis in CD8⁺ T cells cultured in a Transwell system with SiHa-DDP cells with ATP7A knockdown. (K-L) CFSE assay evaluating CD8⁺ T-cell proliferation under Transwell coculture conditions. (M) The conditioned medium (CM) culture system in which CD8⁺ T cells were treated with CM collected from SiHa-DDP cells. (N-P) Flow cytometry analysis of IFN-γ and GzmB expression and apoptosis in CD8⁺ T cells treated with CM from ATP7A-knockdown SiHa-DDP cells (Q-R) CFSE analyses of the proliferation of CD8⁺ T cells exposed to CM.

Figure S6: (A-B) Flow cytometry assay for IFN-γ and GzmB expression in murine murine CD8⁺T cells treated with 5 µM CuCl₂ for 24 h. (C~~,~~-D) Analyze of murine CD8⁺T cell apoptosis after treated with 5 µM CuCl₂ for 24 h. (E~~,~~-F) Proliferation assay of murine CD8⁺T cells after treated with 5 µM CuCl₂ for 24 h. (G~~,~~-H) Flow cytometry analysis of IFN-γ and GzmB expression in murine CD8⁺T cells co-cultured with U14 cells pre-treated with 1 µM TTM for 6 h. (I-~~J~~L) Proliferation and apoptosis assay of murine CD8⁺T cells co-cultured with U14 cells pre-treated with 1 µM TTM for 6 h.

Figure S7: (A) Predicted structural model showing Cu²⁺ coordination within the LCK activation loop near Tyr394. (B-C) Root mean square deviation (RMSD) and Cu²⁺-Tyr394 distance analysis from molecular dynamics simulations. (D) Heatmap of transcription factors correlated with ATP7A expression and prioritized for cisplatin resistance. (E) Kaplan-Meier analysis of overall survival in cervical cancer patients stratified by SOX21 expression. (F-G) Dual-luciferase reporter assays assessing ATP7A promoter activity in SiHa-DDP cells using constructs containing full-length (FL), F1, and F2 fragments and mutant variants (Mut1 and Mut2). (H) ChIP-qPCR assay examining SOX21 enrichment at the ATP7A promoter region in SiHa-DDP cells, with the NC region serving as a negative control. (I) Representative gating strategy for the identification of tumor-infiltrating CD8⁺T cells in flow cytometric analysis.
