# Supplementary material for: ATP7A-mediated copper ion efflux reprogrammes tumour immunity and promotes cisplatin resistance in cervical cancer
Source: Cell Death Dis. 2026 Jul 10;17(1):859. doi: 10.1038/s41419-026-09082-5 (PMC13639005; doi:10.1038/s41419-026-09082-5)
Supplement: Supplementary file 3 [file 41419_2026_9082_MOESM2_ESM.docx]

Table S1: Primer sequences used for qPCR

| Primer name | Sequence (5′–3′) |
| --- | --- |
| ATP7A-Site1.F | GGATCTTTTATGTTACATCTTG |
| ATP7A-Site1.R | CCCCCACGACAAAAAGGGAAAC |
| ATP7A-Site2.F | TCGCTTTTGTGTGGGGGC |
| ATP7A-Site2.R | GGCCGACACGCAGCCTCG |
| NC.F | TTACAGGCATGAGACACCG |
| NC.R | CACGATCAAGAAACTACAAAAA |
| ATP7A-1.F | AGGATCGGTCAGCAAGTCAC |
| ATP7A-1.R | ACCAGCCTCCGAAAAAACTGT |
| SOX21-1.F | GCACAACTGCGAGATCAGCA |
| SOX21-1.R | CCGGGAAGGCGAACTTGTC |
| SOX21-2.F | AACTCGGAGATCAGCAAGCG |
| SOX21-2.R | ACCGGGAAGGCGAACTGT |
| ATP7A-2.F | GGACCACCACTTTGCAACTC |
| ATP7A-2.R | TGGTTGCCAGCACAATCAGT |
| GAPDH-F | GTCAGTGGTGGACCTGACCT |
| GAPDH-R | TGAGCTTGACAAAGTGGTCG |

Table S2: Baseline characteristics of NHANES participants

| Variable | Total |
| --- | --- |
| Age, Mean (SE) | 63.38 (0.92) |
| Average energy intake (2-day mean), Mean (SE) | 1988.50 (45.29) |
| Body Mass Index (BMI), Mean (SE) | 28.49 (0.43) |
| Sex, n (%) |  |
| Male | 230 (50.11) |
| Female | 229 (49.89) |
| Smoking status, n (%) |  |
| Never smoker | 202 (44.01) |
| Former smoker | 169 (36.82) |
| Current smoker | 88 (19.17) |
| Smoking history, n (%) |  |
| No | 202 (44.01) |
| Yes | 257 (55.99) |
| Alcohol consumption, n (%) |  |
| No | 122 (28.24) |
| Yes | 310 (71.76) |
| Alcohol intake category, n (%) |  |
| Low | 173 (65.77) |
| Moderate | 67 (25.48) |
| High | 23 (8.75) |
| Physical activity level, n (%) |  |
| Low | 215 (46.84) |
| High | 244 (53.16) |
| Race/ethnicity, n (%) |  |
| Mexican American | 37 (8.06) |
| Non-Hispanic White | 298 (64.92) |
| Non-Hispanic Black | 65 (14.16) |
| Other race/ethnicity | 59 (12.86) |
| Marital status, n (%) |  |
| Married/living with partner | 279 (60.92) |
| Separated/divorced/widowed | 149 (32.53) |
| Never married | 30 (6.55) |
| Poverty Income Ratio (PIR), n (%) |  |
| Low (<1.3) | 111 (26.49) |
| Middle (1.3–3.5) | 179 (42.72) |
| High (≥3.5) | 129 (30.79) |

Gene Symbol

SLC31A1

SLC31A2

ATOX1

CCS

COX17

ATP7A

ATP7B

SCO1

SCO2

COX11

MT1A

MT2A

MT1E

MT1X

COMMD1

GLRX

CP

COX19

Table S3: Copper homeostasis-related gene set used for AUCell activity scoring

Table S4: Cisplatin response-related gene set used for singscore scoring

| Group | Gene Symbol |
| --- | --- |
| Cisplatin resistance-up | ERCC1 |
|  | ERCC4 |
|  | XPA |
|  | XPC |
|  | RAD51 |
|  | BRCA1 |
|  | BRCA2 |
|  | XRCC1 |
|  | POLH |
|  | REV1 |
|  | FANCD2 |
|  | ATP7B |
|  | ABCC2 |
|  | ABCB1 |
|  | ABCG2 |
|  | GSTP1 |
|  | GSR |
|  | GCLC |
|  | GCLM |
|  | SLC7A11 |
|  | MT1A |
|  | MT1E |
|  | MT2A |
|  | BCL2 |
|  | MCL1 |
|  | ATOX1 |
| Cisplatin resistance-down | SLC31A1 |
|  | BAX |
|  | PMAIP1 |
